# Structural implications of lipoarabinomannan glycans from global clinical isolates in diagnosis of *Mycobacterium tuberculosis* infection

**DOI:** 10.1016/j.jbc.2021.101265

**Published:** 2021-09-30

**Authors:** Prithwiraj De, Anita G. Amin, Danara Flores, Anne Simpson, Karen Dobos, Delphi Chatterjee

**Affiliations:** Mycobacteria Research Laboratories, Department of Microbiology, Immunology and Pathology, Colorado State University, Fort Collins, Colorado, USA

**Keywords:** lipoarabinomannan, acylation, tuberculosis, diagnosis, *Mycobacterium tuberculosis*, LAM, lipoarabinomannan, MS, mass spectrometry, Mtb, *Mycobacterium tuberculosis*, MTX, methylthioxylose, NMR, nuclear magnetic resonance, POC, point-of-care, TB, tuberculosis, UPLC, ultraperformance liquid chromatography

## Abstract

In *Mycobacterium tuberculosis* (Mtb), surface-exposed Lipoarabinomannan (LAM) is a key determinant of immunogenicity, yet its intrinsic heterogeneity confounds typical structure–function analysis. Recently, LAM gained a strong foothold as a validated marker for active tuberculosis (TB) infection and has shown great potential in new diagnostic efforts. However, no efforts have yet been made to model or evaluate the impact of mixed polyclonal Mtb infections (infection with multiple strains) on TB diagnostic procedures other than antibiotic susceptibility testing. Here, we selected three TB clinical isolates (HN878, EAI, and IO) and purified LAM from these strains to present an integrated analytical approach of one-dimensional and two-dimensional Nuclear Magnetic Resonance (NMR) spectroscopy, as well as enzymatic digestion and site-specific mass spectrometry (MS) to probe LAM structure and behavior at multiple levels. Overall, we found that the glycan was similar in all LAM preparations, albeit with subtle variations. Succinates, lactates, hydroxybutyrate, acetate, and the hallmark of Mtb LAM-methylthioxylose (MTX), adorned the nonreducing terminal arabinan of these LAM species. Newly identified acetoxy/hydroxybutyrate was present only in LAM from EAI and IO Mtb strains. Notably, detailed LC/MS-MS unambiguously showed that all acyl modifications and the lactyl ether in LAM are at the 3-OH position of the 2-linked arabinofuranose adjacent to the terminal β-arabinofuranose. Finally, after sequential enzymatic deglycosylation of LAM, the residual glycan that has ∼50% of α−arabinofuranose -(1→5) linked did not bind to monoclonal antibody CS35. These data clearly indicate the importance of the arabinan termini arrangements for the antigenicity of LAM.

Lipoarabinomannan (LAM) is a heterogeneous lipoglycan, a major component of the cell wall of mycobacteria (1, 2). It is characterized by three distinct structural domains: (i) a phosphatidylinositol anchor, (ii) a mannan backbone, and (iii) several arabinan antennas emanating from the mannan backbone. The nonreducing end of the arabinan component of LAM can be left unmodified to form AraLAM in *Mycobacterium chelonae* and capped with phosphoinositol (PI) to form PILAM in *Mycobacterium smegmatis* (3). In the pathogenic mycobacterial species (*e.g.*, *Mtb*, *Mycobacterium leprae*, *Mycobacterium avium*), the termini of the arabinan are further substituted with short mannan caps (di- or tri-saccharide) (4, 5), resulting in the mannosylated LAM (ManLAM). The mannose cap of ManLAM has been implicated in interaction with the mannose receptor (6) and dendritic cell-specific ICAM-3 grabbing nonintegrin (DC-SIGN) to mediate the inhibition of proinflammatory responses such as IFN-γ and IL-12 cytokine secretion (7, 8). On the other hand, PILAM induces a strong proinflammatory response in macrophages, presumably mediated by the PI residue, since the uncapped AraLAM of *M. chelonae* does not induce cytokine secretion or apoptosis of macrophages (6). The linear terminus Ara_4:_ (β-D-Ara*f*-(1→2)-α-D-Ara*f*-(1→5)-α-D-Ara*f*-(1→5)- α-D-Ara*f*) (Fig. 1*A*) and a branched terminus Ara_6_:([β-D-Ara*f*-(1→2)-α-D-Ara*f*-(1-)_2_ →3, and→5]-α-D-Ara*f*-(1→5)-α-D-Ara*f*) (Fig. 1*B*) have been shown to be the epitopes recognized by anti-LAM monoclonal antibodies (mAb) (9, 10, 11, 12, 13) irrespective of their capping functions. However, point-of-care (POC) methods to detect LAM using immunoassays have been stagnant due to suboptimal sensitivity and are limited to only active TB diagnosis with severe disease and those persons with coinfection with HIV (14, 15, 16) although it has been clearly shown that LAM is present in active TB cases without HIV coinfection (17, 18). We and the others actively pursuing the field have speculated that these disappointing results with the POC methods may be due to the antibodies used in the immunoassays that are generally raised against one common laboratory strain (10, 11, 19, 20), and demographical strain specific anti-LAM antibodies will be required for use in TB endemic countries. We recently reported that LAM from Mtb SA161, same strain infecting mouse lungs as well as a TB+ HIV patient's urine, had non reducing ends that are mostly a linear stretch of 5xAra*f*s: β-D-Ara*f*-(1→2)-α-D-Ara*f*-(1→5)-α-D-Ara*f*-(1→5)-α-D-Ara*f*-(1→5)-α-D-Ara*f*- (Fig. 1*C*) and a lack of the branched Ara_6_ (B). In addition, covalent acyl modifications such as succinyl monoester at the 3-hydroxyl (C3) of the 2-linked arabinose (the penultimate Ara*f* at the nonreducing end) were also reported (21). To this end, succinates have been reported in LAM and arabinogalactan as minor components in Mtb, *M.smegmatis*, *M. leprae* and *Mycobacterium bovis*, BCG, and located on variable locations (22).

**Figure 1:**
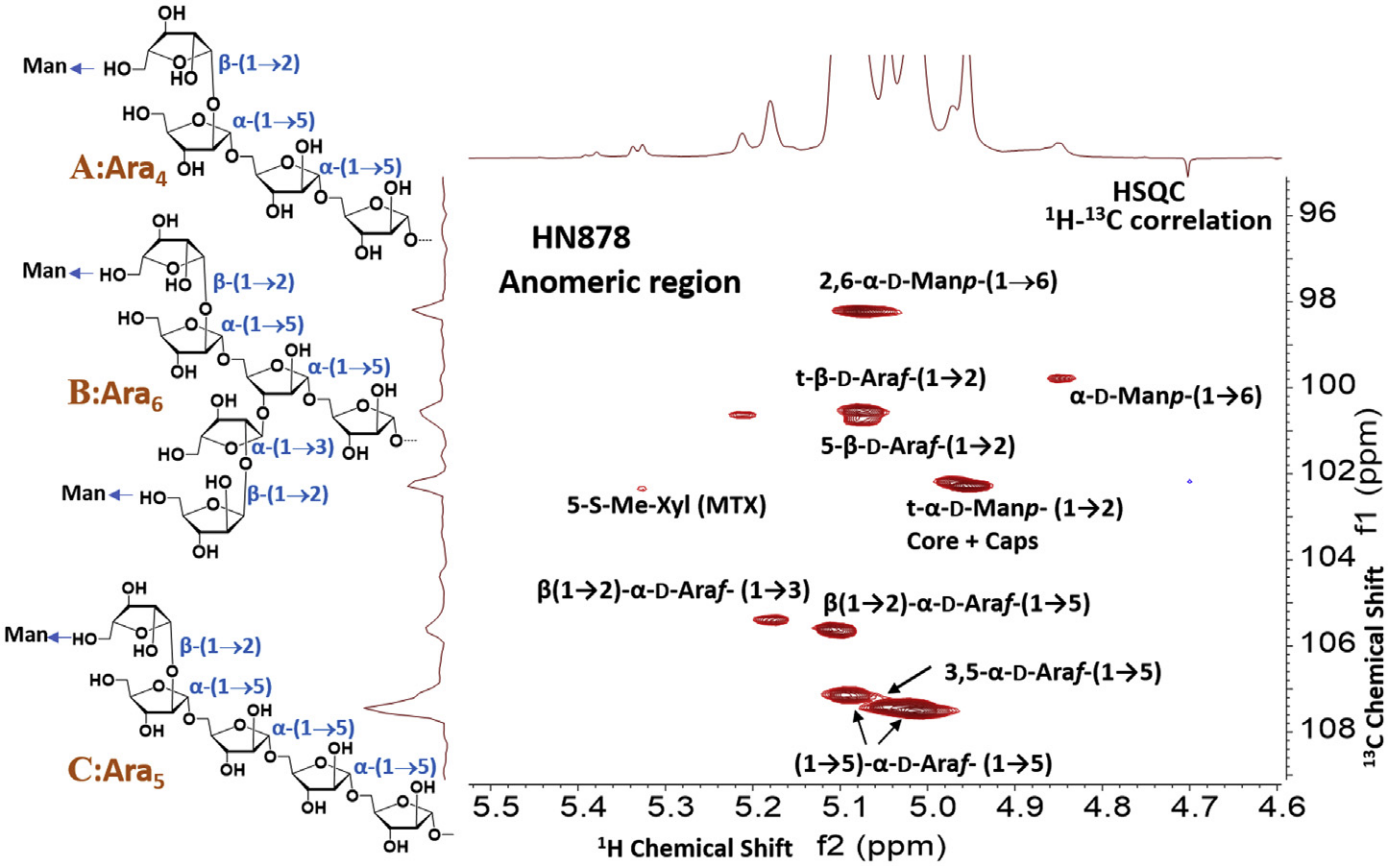
**The HSQC NMR (**^**1**^**H-**^**13**^**C 2D correlation) spectrum of HN878 LAM (Anomeric region; representative)**. NMR was performed in D_2_O at 25 °C. The variety of glycosidic linkages involving arabinofuranoses, mannopyranoses, and MTX (5-SMe-Xyl*f*) were clearly visible. The structure of (*A*) Ara_4_, (*B*) Ara_6_, and (*C*) Ara_5_ has been shown.

The human adapted *Mycobacterium tuberculosis* complex (MTBC) exhibits a strong phylogeographical population structure, with some lineages occurring globally and others showing a strong geographical restriction (23, 24). Among these lineages L2 and L4 are the most widespread globally, with L2 dominating in East Asia. L1 and L3 occur mainly in regions around the Indian Ocean. L5 and L6 are highly restricted to West Africa, whereas L7 is almost exclusively found in Ethiopia. Geographic location can introduce variability for TB screening because of heterogeneity in TB strain or clade prevalence. Our hypothesis was that Mtb clinical isolates have a wide spectrum of virulence, which is lineage-associated, modulates host immune response, and determines bacterial load in patients with pulmonary tuberculosis. The laboratory passaged strain H37Rv exhibits intermediate virulence, causing ∼50% macrophage lysis. We took this as a reference strain in this study.

Based on this information, in our work of global LAM characterization, we selected Mtb strains EAI from L3, IO from L1 and HN878 from L2 compared with H37Rv. LAM was purified in sufficient quantities from each strain to perform thorough analyses focusing primarily on NMR at first so that all features can be assessed in the native molecule, followed by enzymatic digestion and mass spectrometry analyses on released oligoarabinofuranosides. Analyses were carried out without any downstream derivatization to maintain the integrity of all substitution/s.

The objectives of this study were to map the LAM phenotype in bacterial strains that cause TB disease in TB endemic geographical areas and examine whether any epidemiologically relevant structural characteristics were associated with those strains. Our study provides a comprehensive systematic analysis of the evidence for diversity in LAM in particular in bacterial strains that are of clinical relevance.

## Results

### Isolation of LAM from clinical isolates

The TB clinical isolates represent three geographically distinct lineages (as referenced in (25)) wherein, HN878 (East Asia lineage), T17-IO (The Philippines/Rim of the Indian Ocean lineage) East African-Indian 91-0079-EAI (India and East Africa lineage) represent the most globally predominant lineages outside of those typed to the Europe and Americas lineage. The laboratory-type strain Mtb H37Rv was used as a reference strain and represents the Europe and Americas lineage. We investigated the intact LAM by extensive 1D and 2D NMR spectroscopy to find out differences in glycosidic linkages as well as small-molecule modifications. The findings were supported by mass spectroscopy on the enzyme-digested LAM terminal-arabinan fragments since these arrangements were presumed to be antibody binding structures.

### Overall sugar network in LAM

1D-proton NMR showed no marked difference among LAM isolated from HN878, EAI, IO compared with laboratory strain H37Rv (Fig. S1). Among these LAMs, a larger sugar domain in comparison to the fatty acyl region was apparent for RvLAM as estimated by integrated peaks. ^1^H-^13^C correlation spectra (HSQC full spectrum presented in Fig. S2, *A–D*) revealed that all LAM has similar glycosidic linkages as reported in the literature (13, 26). The representative HSQC spectrum of the HN878 LAM (focusing on the anomeric region) (Fig. 1) revealed the presence of all possible linkages expected from MtbLAM. The anomeric protons of Ara*f*s are assigned to: 5-linked arabinofuranoses, α-D-Ara*f*- (1→5) at δ 5.02, 5.09 (H-1), δ 107.5, 107.2 (C-1) ppm; 3,5-linked arabinofuranoses, *i.e.*, α-D-Ara*f* -(1→5)- α-D-Ara*f* –[α-D-Ara*f* -(1→3)]-(1→5) at δ 5.07 (H-1), δ 107.2 (C-1) ppm 2-linked Ara*f*; β-D-Ara*f* (1→2)- α-D-Ara*f* -(1→5) at δ 5.10 (H-1), δ 105.7 (C-1) ppm, 2-linked Ara*f* (branch); β-D-Ara*f* (1→2)- α-D-Ara*f* -(1→3) at δ 5.18 (H-1), δ 105.5 (C-1) ppm and terminal nonmannose-capped and mannose-capped arabinofuranoses; t- β-D-Ara*f*- (1→2) and α-D-Man-(1→5)- β-D-Ara*f*-(1→2) at δ 5.07 (H-1), δ 100.6, 100.8 (C-1) ppm, respectively. The anomeric protons of Man*p*s are assigned to: 2,6-linked Man*p*; & α-D-Man*p*-(1→6)-Man*p*-[α-D-Man*p*-(1→2)]-(1→6) at δ 5.07 (H-1), δ 98.4 (C-1) ppm, 6-linked Manp; Man*p*-α(1→6) at δ 4.85 (H-1), δ 99.8 (C-1) ppm, and terminal as well as 2-linked Man*p* (mannan core and mannose caps (5, 13)); α-D-Man*p*- (1→2) at δ 4.95, 4.97 (H-1), δ 102.4 (C-1) ppm respectively. Additionally, anomeric proton/carbon for MTX (5-SMe-α-D-Xyl*f*) was also visible at δ 5.32 (H-1), δ 102.5 (C-1) ppm as indicated in Figure 1. MTX was further characterized using ^1^H-^1^H correlation (TOCSY) spectroscopy. TOCSY NMR spectrum of LAM from HN878, IO and H37Rv is presented in the Figure S3, *A–C*.

### Acylation substitutions in LAM

**Figure 2:**
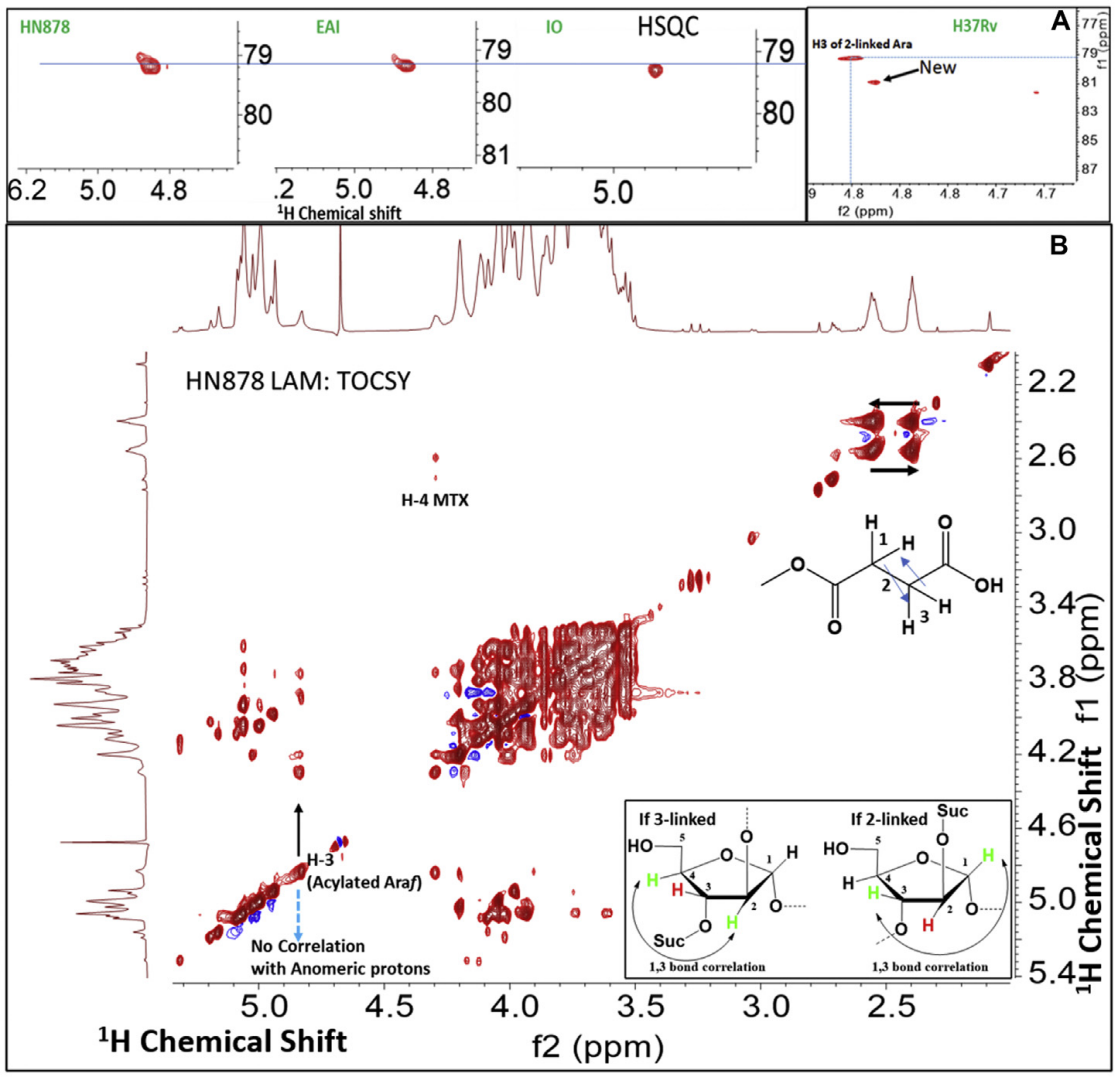
**The location of major acyl functionalities, such as succinyl monoester, on a particular Ara*f*-units of four-LAM samples**. *A*, the HSQC NMR spectrum of LAM samples (HN878, EAI, IO, and H37Rv respectively) showing the particular region of deshielded Ara*f*-ring-proton-carbon (δ 4.87 (H-3), δ 79.2 (C-3). Only H37Rv, a laboratory strain, has an additional weak peak in the region (δ 4.85 (H), δ 81.1 (C) suggests additional location of acyls. *B*, the zTOCSY NMR (^1^H-^1^H correlation) spectrum (D_2_O, 25 °C) of HN878 LAM showing cross peaks at δ 2.52 and δ 2.48 ppm, assigned as -O-C(O)-CH_2_-CH_2_-C(O)-OH (succinate-type) linkage. The proton at δ 4.87 (H-3) did not show any 1,3-bond correlation to any anomeric proton confirming it to be H-3 and not H-2 in Ara*f*-residue (*inset*).

Succinyl residues are linked through an ester bond and only one set of acylated ring proton was observed in each of the HSQC spectra for HN878, EAI, and IO-LAM (Fig. 2*A*). The cross peak at δ 4.85 ppm (^1^H) and δ 79.2 ppm (^13^C) was attributed to the H3/C3 of an arabinose ring. In fact, all the acyl functionalities should be linked through this proton unless they occupy the primary –OH groups. However, the absence of any downfield proton that could be attributed to H5 of Ara*f* or H6 of Man*p* supports this finding. In addition, the absence of any anomeric proton in 1,3-bond correlation (in TOCSY) with the acylated ring proton ruled out acylation at the 2-position of Ara*f* (Fig. 2*B*). RvLAM on the other hand was found to have two sets of acylated ring protons (Fig. 2*A*) with different intensities. The major cross peak was the H3/C3 of the Ara*f* ring as found in all four LAM, and the minor cross peak was found at δ 4.83 ppm (^1^H) and δ 81.1 ppm (^13^C). Unfortunately, the weak peak could not affirm 1,3-bond correlation (TOCSY) with any anomeric proton, in the event the succinate was at the 2-position. The presence of a succinyl residue at the 2-position of 3,5-linked Ara*f* was reported earlier in arabinogalactan isolated from Mtb H37Rv (27).

**Figure 3:**
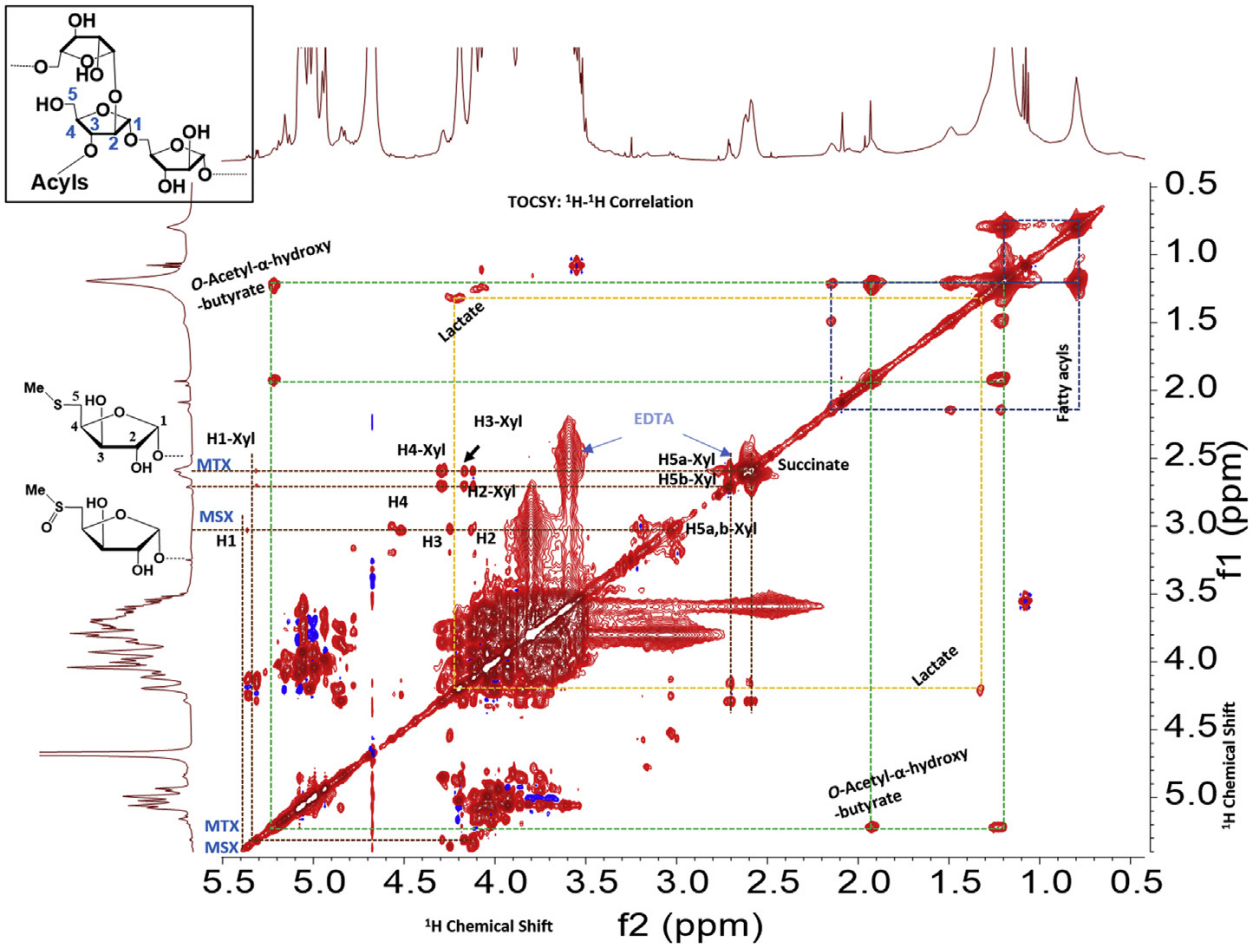
**The TOCSY (**^**1**^**H-**^**1**^**H correlation) NMR spectrum (D**_**2**_**O, 25 °C) of EAI-LAM**. Acyl residues such as succinate, α-hydroxybutyrate, α-acetoxybutyrate along with lactyl-group and methyl thioxylose (MTX) and methyl sulfoxyxylose (MSX) are shown.

The TOCSY of HN878 LAM (Fig. 2*B*) showed two sets of protons corroborating to a -C(O)-CH_2_-CH_2_-C(O)- structure unequivocally establishing a succinyl monoester residue. For confirmation, additional support was sought from enzymatic digestion of LAM. As a representative structure, the extracted ion chromatogram (LC/MS of enzyme digested LAM arabinan) showed same retention time for the ion *m/z* 1101.34 [M-H]¯ corresponding to Suc_1_Man_2_Ara_5_ for all the 3× LAM species (HN878, EAI and IO). This indicated that the monosuccinylated Man_2_Ara_5_ has a similar structure in all LAM. MS-MS was performed on this ion to reveal the sequence (Fig. S4). This showed that Ara_5_ structures are always linear in LAM (21). The analysis also supports the location of succinate at the 3-position of 2-linked Ara*f*, as shown by NMR and described before. One interesting aspect was noted about the succinyl residues in EAI and IO-LAM. The ^1^H-chemical shifts of the succinyl protons merged at δ 2.6 ppm giving an overlapping signal for 4× protons instead of usual δ 2.4 and δ 2.6 ppm (Figs. 3 and S3, *A–C*). Possibly, a charge-based association of succinate with EDTA (used during purification; as evident in NMR) is responsible for this shift of peaks.

The TOCSY NMR of EAI-LAM (Fig. 3) clearly revealed that there were other unique substitutions present as covalent modifications of LAM. The 1-3-bond correlation of protons at δ 4.2 and δ 1.3 ppm suggested a lactate-like structure and correlations of protons at δ 4.1, δ 2.1, and δ 1.2 ppm suggested a higher homolog of lactate (Fig. 3), such as α-hydroxy butyrate-like structure. In addition, correlations of protons at δ 5.2, δ 1.9, and δ 1.2 ppm suggested an α-*O*-acetyl butyrate-like structure. The downfield shift of the α-proton of α-hydroxy butyrate from δ 4.1 to δ 5.2 ppm was attributed to the electron withdrawing effect of the *O*-acetyl group. These variations were less prominent in IO-LAM and undetectable in HN878 LAM and RvLAM.

### Confirmation of lactyl ether using LC/MS-MS

**Figure 4:**
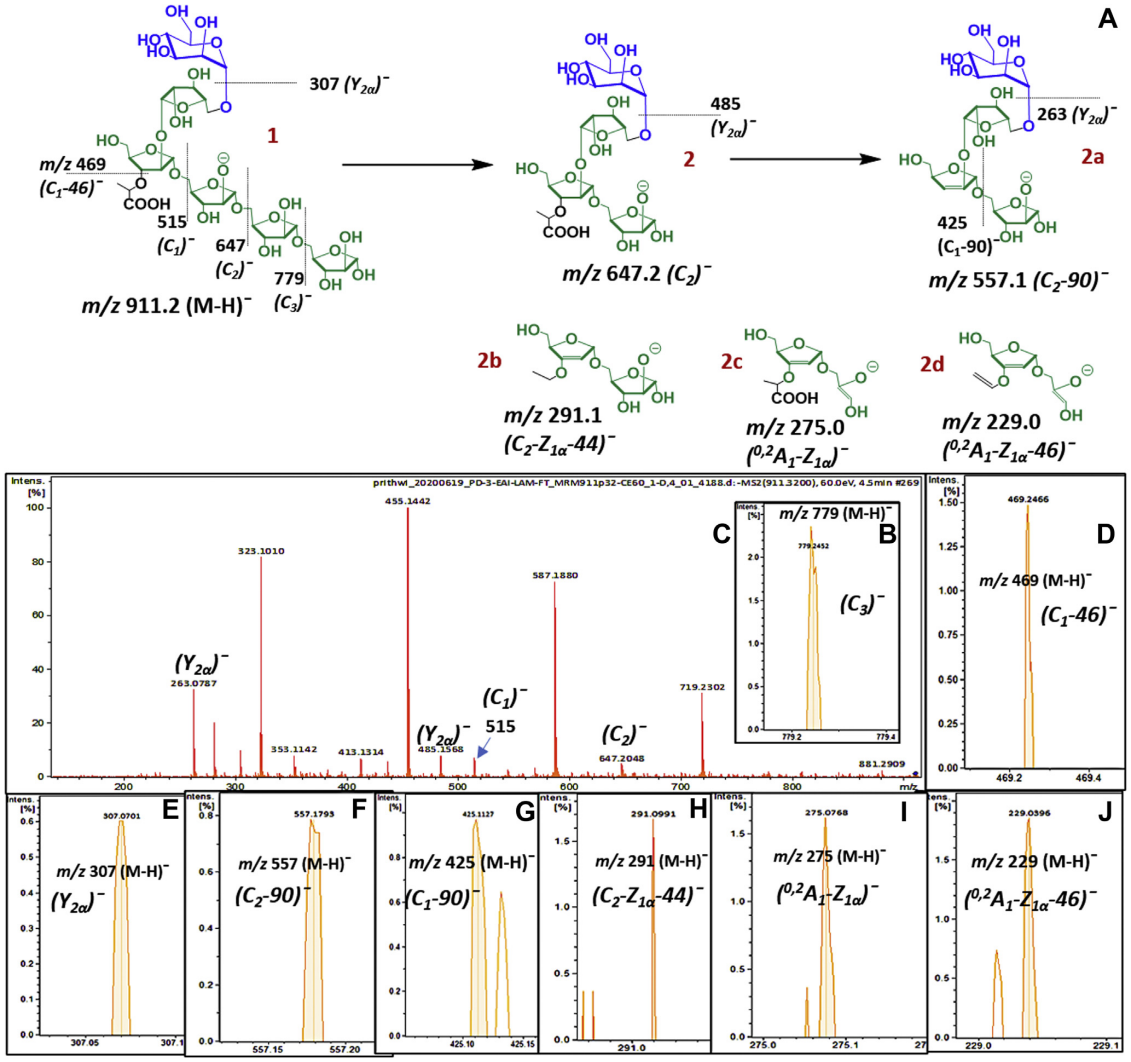
**Lactyl group; MS-MS analysis (negative ion, 60 ev, EAI-LAM; m/z 911.29 [M-H]¯) of Lactyl-Man**_**1**_**Ara**_**5**_**showing it is ether linked and its position**. *A*, pathway of LC/MS-MS fragmentation for Lactyl-Man_1_Ara_5_. *B–J*, explained ions from the LC/MS-MS fragmentation, in support of structural assignments. Explained ions from the LC/MS-MS fragmentation, in support of structural assignments.

Lactyl group was identified in all the LAM samples from four different Mtb species by LC-MS. Although, we had reported earlier (21) that alkali-hydrolysis did not remove lactyl group in *in vivo* LAM, at that time, because of lack of material, MS-MS analysis on enzyme-released fragments was not possible. Herein, analysis was achieved on *m/z* 911.29 (presumably lactyl Man_1_Ara_5_ [M-H]^¯^; 1; Fig 4*A*) by LC/MS-MS on EAI-LAM. The ion at *m/z* 911.29 [M-H]^¯^can also arise from monoacetyl Man_2_Ara_4_. However, after sequential digestion with α-mannosidase followed by *endo*arabinanase, no acetyl was found associated with Ara_4_ residues in case of the EAI LAM. Three consecutive losses of 132 Da (each) were accounted for three unsubstituted pentoses (Ara*f*) at the reducing end. The corresponding ions were found at *m/z* 779.2 [M-H]^¯^ (C_3_)¯(Fig. 4*B*), *m/z* 647.2 [M-H]^¯^ (C_2_)¯ (2; Fig. 4*C*; ), and *m/z* 515.1 [M-H]^¯^ (C_1_)¯(*2a*; Fig. 4*C*) respectively. The ion (C_1_)¯ underwent a specific fragmentation that is associated to a loss of 46 Da-(formic acid), at *m/z* 469.2 [M-H]^¯^ (C_1_-46)¯(1; Fig. 4*D*). This fragmentation clearly suggested that a free carboxylic group was present, thereby indicative of the lactyl group attached to LAM *via* ether linkage. The presence of *m/z* 307.0 [M-H]^¯^ (Y_2α_)¯ (1; Fig. 4*E*) indicated that (the loss of) a hexose (Man*p*) is not associated to the lactyl. So, the lactyl group is attached to one of the two Ara*f* units at the nonreducing end. At this point, we also noticed that the ion *m/z* 647.2 [M-H]^¯^ (C_2_)¯ (2; Fig. 4*C*) undergoes some specific cleavages. The loss of 90 Da (72 Da from lactyl group and 18 Da for loss of water), which accounts for the ion at *m/z* 557.1 [M-H]^¯^ (C_2_-90)¯ (Fig. 4*F*), is suggestive of the lactyl group associated to a secondary ring hydroxy-group. Sequential losses of 132 Da (*m/z* 425.1 [M-H]^¯^ (C_1_-90)¯ (2a; Fig. 4*G*) followed by 162 Da (*m/z* 263.1 [M-H]^¯^ (Y_2α_)¯(Fig. 4*C*)) conclude again that the lactyl group is attached to one of the two Ara*f* units at the nonreducing end. The presence of a specific ion at *m/z* 291.1 [M-H]^¯^ (C_2_-Z_1α_-44)¯ (2b; Fig. 4*H*) is indicative of (i) the carboxyl unit lost as carbon dioxide (44 Da) and (ii) neither the mannose cap nor the β-Ara*f* bear lactyl ether. However, the presence of two specific D-type ions at *m/z* 275.0 [M-H]^¯^ (^0,2^A_1_- Z_1α_)¯ (2c; Fig. 4*I*) and *m/z* 229.0 1 [M-H]^¯^ (^0,2^A_1_- Z_1α_-46)¯ (2d; Fig. 4*J*) not only suggests the structural assignment of 2b but also establishes that the lactyl-ether is attached to the 2-linked Ara*f*, which has a 3-position, available for the lactyl substitution.

### Confirmation of α-hydroxy butyrate using LC/MS-MS

**Figure 5:**
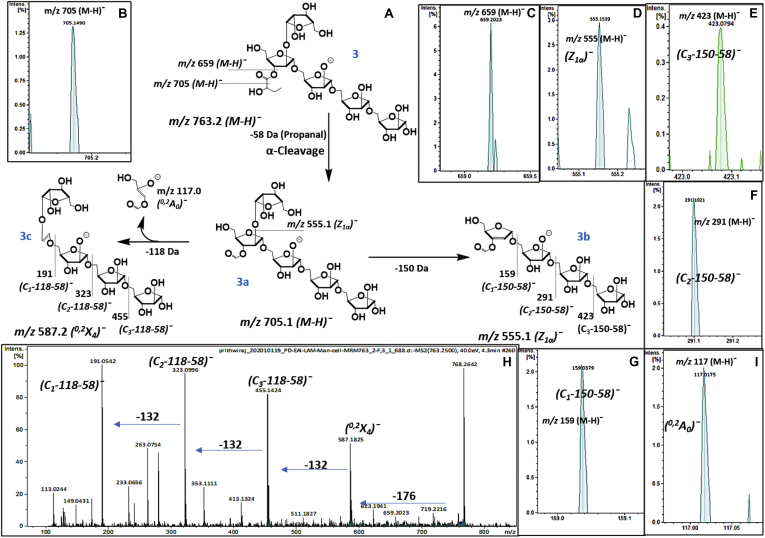
**α-Hydroxy butyrate; MS-MS analysis (negative ion, 40 ev, EAI-LAM, m/z 763 [M-H]¯) of α-hydroxy butyryl-Ara**_**5**_**(obtained after sequential enzymatic digestion) showing ester linkage and position**. *A*, pathway of LC/MS-MS fragmentation for α-hydroxy butyryl-Ara_5_. *B–I,* explained ions from the LC/MS-MS fragmentation, in support of structural assignments. Explained ions from the LC/MS-MS fragmentation, in support of structural assignments.

The α-hydroxy butyryl group mainly associated with the linear Ara_5_ and the branched Ara_6_ termini of EAI LAM. This was established by the LC/MS-MS analysis in the negative ion mode. Representative ion at *m/z* 763.2 [M-H]^¯^ (α-hydroxy butyryl Ara_5_; 3; Fig. 5*A*), obtained after sequential enzymatic digestion, revealed a specific neutral loss of 58 Da to produce *m/z* 705.1 [M-H]^¯^ (3a; Fig. 5*B*). This loss was attributed to the loss of propanal (58 Da) produced by the α-cleavage of α-hydroxy butyrate (28). The presence of *m/z* 659.2 [M-H]^¯^ (3; Fig. 5*C*) *via* a neutral loss of 104 Da (86 Da from α-hydroxy butyryl group +18 Da from water) is indicative of the substitution associated to a secondary ring hydroxy-group. The formate-Ara_5_ (*m/z* 705.1 [M-H]^¯^) (3a) underwent further fragmentation with a loss of 150 Da (Ara*f*). This ion at *m/z* 555.1 [M-H]^¯^ (Z_1α_-58)¯ (3b; Fig. 5*D*), accounts for a loss of 18 Da (water) along with cleavage of Ara*f* from the nonreducing end. This assignment was made since C-Z-type fragmentations were found to be dominant during fragmentation pathway. Further loss of 3xAra*f* from 3b the reducing end gave ions at *m/z* 423.0 [M-H]^¯^ (C_3_- Z_1α_ -58)¯ (3b; Fig. 5*E*), *m/z* 291.1 [M-H]^¯^ (C_2_- Z_1α_ -58)¯ (3b; Fig. 5*F*), and *m/z* 159.0 [M-H]^¯^ (C_1_- Z_1α_ -58)¯; (3b; Fig. 5*G*). Formation of these ions also suggests that the 2-linked Ara*f* is also linked to the formyl or α-hydroxy butyryl group. Also, the characteristic cross-ring cleavage (^0,2^X_4_; *m/z* 587 [M-H]; 3c; Fig. 5*H*), a major fragment ion of 2-linked Ara*f*, accounts for a loss of 176 Da (Ara*f* ring contributes 90 Da and α-hydroxy butyryl group contributes 86). Sequential losses of 3× Ara*f* (m/z 455.1 (C_3_-176)¯ (3c; Fig. 5*H*), 323.1 (C_2_-176)¯ (3c; Fig. 5*H*), and 191.0 (C_1_-176)¯ [M-H]^¯^; (3c; Fig. 5*H*) support the presence of the α-hydroxy butyryl group at the 2-linked Ara*f*. The formylated counter ion was also found at *m/z* 117.0 [M-H]^¯^ (^0,2^A_0_)¯ (Fig. 5*I*). Collectively, these specific ions suggest that the α-hydroxy butyrate group is attached to the 2-linked Ara*f*, which has only 3-position, available as a secondary –OH group, for substitution.

### Confirmation of α-acetoxy butyrate using LC/MS-MS

**Figure 6:**
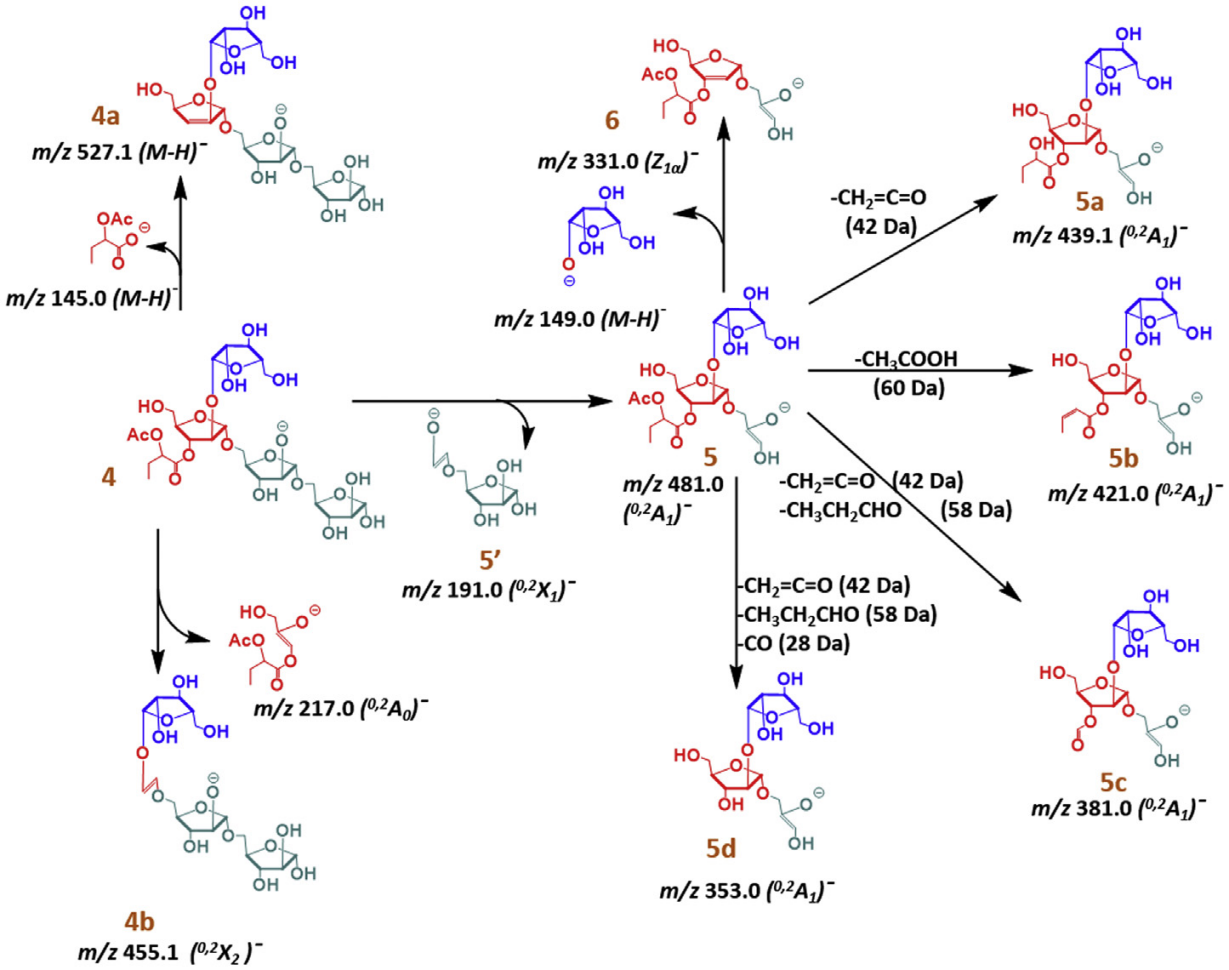
**LC/MS-MS fragmentation of (α-acetoxy butyryl Ara**_**4**_**; m/z 673 [M-H]¯; EAI LAM) (40 eV, negative ion, ESI-collision-induced dissociation) obtained after sequential enzymatic digestions.** Pathway of fragmentation; sequential losses of ketene (42 Da)/acetic acid (60 Da), propanal (58 Da), and carbon monoxide (28 Da) confirm α-acetoxy butyryl group supporting NMR assignments.

We were able to investigate *m/z* 673.2 [M-H]¯ (4; Fig. 6) (α-acetoxy butyryl Ara_4_) by LC/MS-MS experiment. Two consecutive losses of 132 Da each giving ions at *m/z* 541.1 [M-H]¯ (4; Fig. 6, (C_2_)¯) and *m/z* 409.1 [M-H]¯ (C_1_)¯ were observed. This indicated that two Ara*f* units are not acylated. Further loss of 150 Da for a terminal Ara*f* unit produced an ion at *m/z* 259.1 [M-H]¯ (Z_1α_)¯signifying that the substitution could be on the second Ara*f* unit on either terminal. An ion at *m/z* 145.0 [M-H]¯ (Fig. 6), accounting for a loss of 146 Da (α-acetoxy butyric acid) from *m/z* 673.2 [M-H]¯, explains formation of the Z-type ion at *m/z* 527.1 [M-H]¯ (4a). This was suggestive of a substitution associated to secondary -OH of the Ara*f* unit, *i.e.*, 2- or 3-OH. Subsequently, sequential losses of 2× Ara*f* units (132 Da each) from 4a resulted in ions at *m/z* 395.1 [M-H]¯ (C2)¯) and *m/z* 263.0 [M-H]¯ (C1)¯ suggesting that the two successive Ara*f* units at the reducing end are not substituted. The ion at *m/z* 217.0 [M-H]¯ (^0,2^A_0_)¯ suggests a loss of 218 Da (90 from the Ara*f* ring and 128 from the acetoxybutyrate). The corresponding (^0,2^X_2_)¯ ion at *m/z* 455 [M-H]¯ (4b) was also present. These ions signify that the acetoxybutyrate is linked at 3-position of an Ara*f* ring. It also allows limiting the two arabinose units of the nonreducing end where these ^0,2^A-type fragments can occur because the remaining two arabinose units are α-(1→5) linked. Further, sequential losses of 132 Da from 4b gave ions *m/z* 323.0 (C_2_)¯ [M-H]¯ and *m/z* 191.0 (C_1_)¯ [M-H]¯. These ions clearly indicate that the reducing end arabinose units do not carry the butyrate functionality. The cross-ring cleavage giving *m/z* 191.0 [M-H]¯ (^0,2^X_1_)¯ can be produced from the reducing end, and this is crucial for further structural assignments. A series of (^0,2^A_1_)¯ ions such as *m/z* 481.1 [M-H]¯ (5), *m/z* 439.1 [M-H]¯ (5a), *m/z* 421.0 [M-H]¯ (5b), *m/z* 381.0 [M-H]¯ (5c), and *m/z* 353.0 [M-H]¯ (5d) account for the losses of acetate (ketene, 42 Da; acetate, 60 Da), propanal (58 Da, from hydroxy butyrate; α-cleavage), and carbon monoxide (28 Da, from formate). Significantly, further loss of 150 Da, the terminal Ara*f*, from each of these ^0,2^A_1_ ions, produced (Z_1α_)¯ ions at *m/z* 331.0 [M-H]¯, *m/z* 289.0 [M-H]¯, *m/z* 271.0 [M-H]¯, *m/z* 231.0 [M-H]¯, and *m/z* 203.0 [M-H]¯ respectively. These ions decisively establish that the terminal β-Ara*f* is not substituted. Furthermore, it is conclusive that the α-acetoxybutyryl ester is at the 3-position of the 2-linked Ara*f*. Chromatograms of all the ions are presented in the Figure S5, *A* and *B*.

### Confirmation of MTX and MSX using NMR and LC/MS-MS

The presence of methylthioxylose (MTX) residue is evident in the NMR spectra of all LAM preperations-HN878, EAI, IO, and H37Rv. The correlation (TOCSY) of H5a and H5b, the diastereotopic protons, (δ 2.6 and 2.7 ppm) with δ 4.12 (H2), δ 4.18 (H3), δ 4.31 (H4) ppm respectively confirm (26, 29) the presence of MTX. In addition, the sulfoxy variation of MTX, *i.e.*, methylsulfoxylose (MSX) was also found to be present. The presence of MTX/MSX in relatively high NMR peak intensity signifies that there could be multiple sites that bear MTX and/or MSX. So far, it has been shown that MTX in LAM is present one per molecule of LAM (26). Our current observation indicates otherwise.

We had reported earlier that following digestion with *endo*arabinanase and LC/MS of released ararbinan fragments of LAM, low-intensity ions corresponding to MTXMan_1_Ara_5_ (*m/z* 1001.30 [M-H]^¯^) were obtained overlapping with Man_2_Ara_5_ (*m/z* 1001.32 [M-H]^¯^) in LAM (21). Such a small difference in *m/z* values coupled with low abundance made it difficult to unambiguously identify MTX. However, in this present study, in all LAM samples we found *m/z* 1017.31 [M-H]^¯^) and 1149.34 [M-H]^¯^ (Fig. S6) as abundant *endo*arabinanase released arabinan termini. These ions were attributed to MSXMan_1_Ara_5_ and MSXMan_1_Ara_6_, respectively. The 16 Da increment in mass from corresponding MTX-analogs (*m/z* 1001.30 [M-H]^¯^ for MTXMan_1_Ara_5_ and 1133.33 [M-H]^¯^ for MTX Man_1_Ara_6_) supports the presence of sulfoxide functionality.

**Figure 7:**
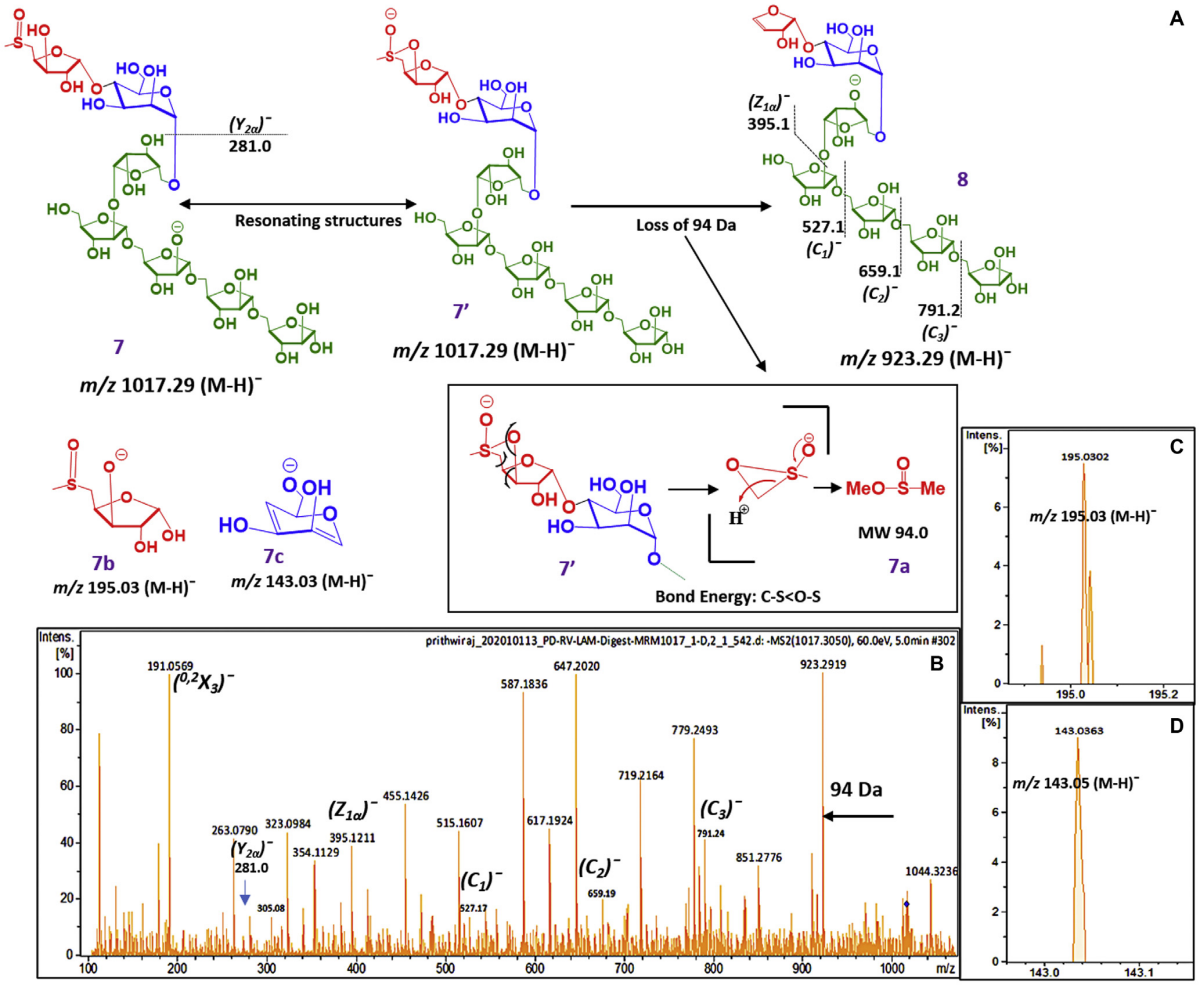
**MSXMan**_**1**_**Ara**_**5**_**; MS-MS analysis (negative ion, 60 ev, EAI-LAM) of MSXMan**_**1**_**Ara**_**5**_**(*m/z* 1017.29 [M-H]¯) (obtained after one-step digestion with arabinanase) showing its position**. *A*, pathway of LC/MS-MS fragmentation for MSXMan_1_Ara_5_. *B–D*, explained ions from the LC/MS-MS fragmentation, in support of structural assignments.

The ion for MSXMan_1_Ara_5_ (7; Fig. 7*A*; *m/z* 1017.29 [M-H]^¯^) can exist in one of the resonating structures 7′ (30). This structure (7′) has a bridging sulfoxide linkage with the 3-OH group of xylose. The bridging sulfoxide (7′) possibly undergoes rearrangement, and an elimination of methanesulfinic acid methyl ester (7a; 94 Da) takes place (inset). This elimination is attributed to the formation of the major ion at m/z 923.29 [M-H]^¯^ (8; Fig. 7*B*). Subsequently, sequential loss of 4× Ara*f* (132 Da each) confirms the presence of methylsulfoxyxylose unit at the nonreducing end. The ion at *m/z* 195.03 [M-H]^¯^ (7b; Fig. 7*C*) confirms that the sulfoxide unit is attached to a pentose (Xyl*f*). However, the formation of *m/z* 143.03 [M-H]^¯^ (7c; Fig. 7*D*) can be attributed to a D-type cleavage to a Man*p* unit. This indicates that the MTX/MSX unit is linked to Man*p* either of the 2-, 3-, or 4- hydroxyl group. Previous work showed that it is linked to (1→4) α-D-Man*p* (31)

### Overall abundance of LAM arabinan termini

**Figure 8:**
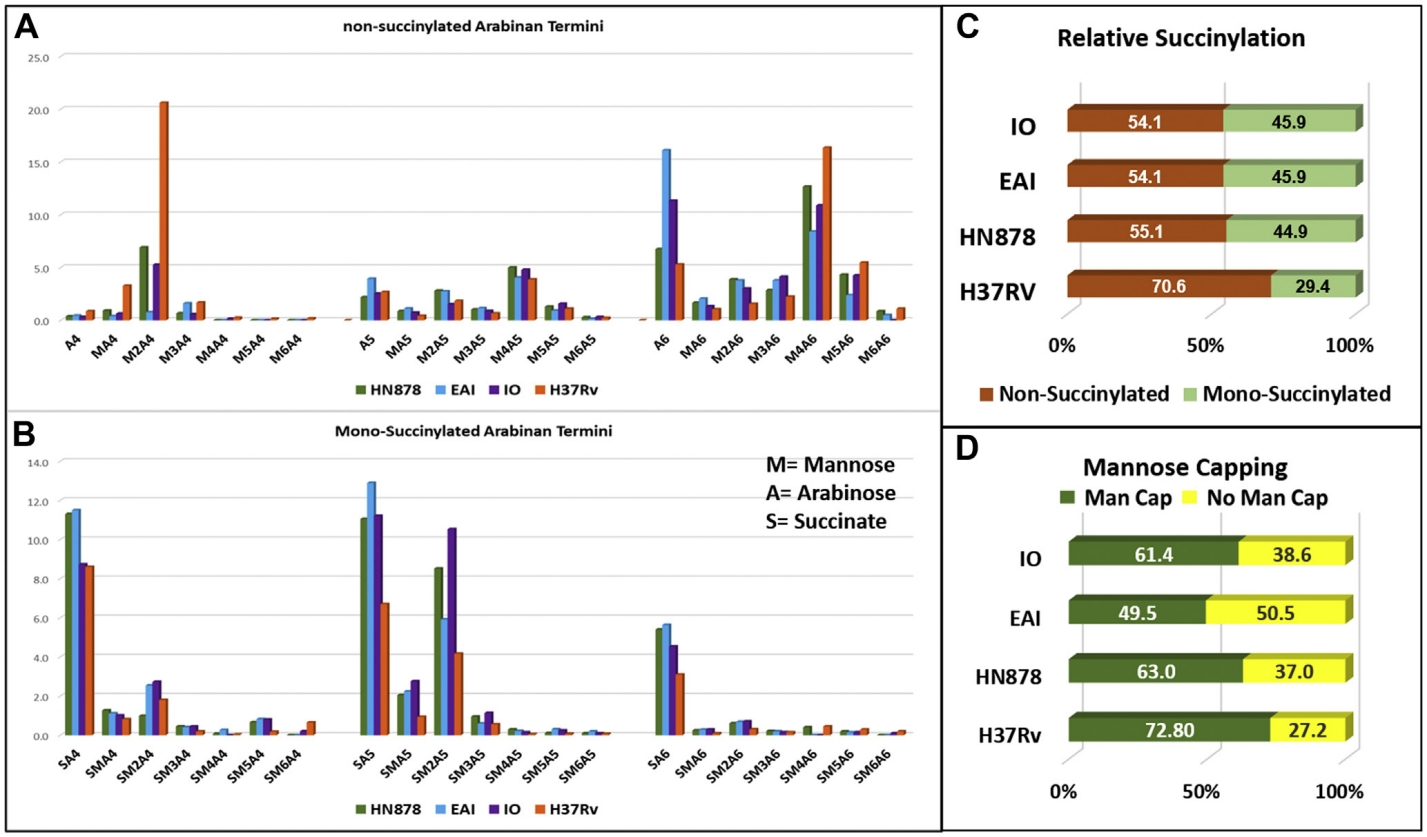
**The LC/MS (negative ion) analysis (normalized) of *endo*arabinanase released LAM arabinan terminus from all 4× LAM samples (HN878, *green*; EAI, *blue*; IO, *purple*; H37Rv, *orange*).***A*, all have Ara_6_ (EAI > IO > HN878 > H37Rv) and its mannose capped variations that are not succinylated. *B*, it appears that four termini (Ara_4_, Ara_5_, Man_2_Ara_5_, and Ara_6_) bear majority of the succinates. *C*, relative overall succinylation (∼45%) was found to be similar in LAM (arabinan termini; Ara_4_, Ara_5_, Ara_6_) from clinical isolates and considerably less in LAM from H37Rv (∼30%). *D*, relative overall mannose-capping of Ara_4_, Ara_5_, Ara_6_ was observed in the order: H37Rv > HN878 > IO > EAI. For simplicity and relevance to the nonreducing end, we restricted our analysis for Ara_4_, Ara_5_, Ara_6_ as released by the enzyme; higher arabinan (Ara_7_–Ara_9_) was also visible in LC/MS with lower abundances.

The LC/MS profile of *endo*arabinanase released LAM arabinan termini revealed that all four LAM samples have abundant Ara_6_ (EAI > IO > HN878 > H37Rv) and its mannose capped variations that are not succinylated (Fig. 8*A*). Two most prominent motifs are Ara_6_ and Man_2_Ara_6_ (H37Rv > HN878 > IO > EAI). Among the monosuccinylated versions, it seemed that the linear Ara_4_ and Ara_5_ with and without mannose capping dominated, although one needs to be cautious that this relative quantification is based on ion intensity only and is not absolute. It appears that four termini (Ara_4_, Ara_5_, Man_2_Ara_5_, and Ara_6_) bear the succinates (Fig. 8*B*). In addition, relative succinylation (Fig. 8*C*) estimated from LC/MS ion abundance analysis reveals that HN878, EAI- and IO-LAM have ∼45% succinylated termini (green/orange segments). Based on the LC/MS ion abundance, Figure 8*D* showed that EAI-LAM has approximately 10–12% less Man capping (green/yellow segment) compared with the LAMs from three other strains in this study.

### Sequential enzymatic digestion

**Figure 9:**
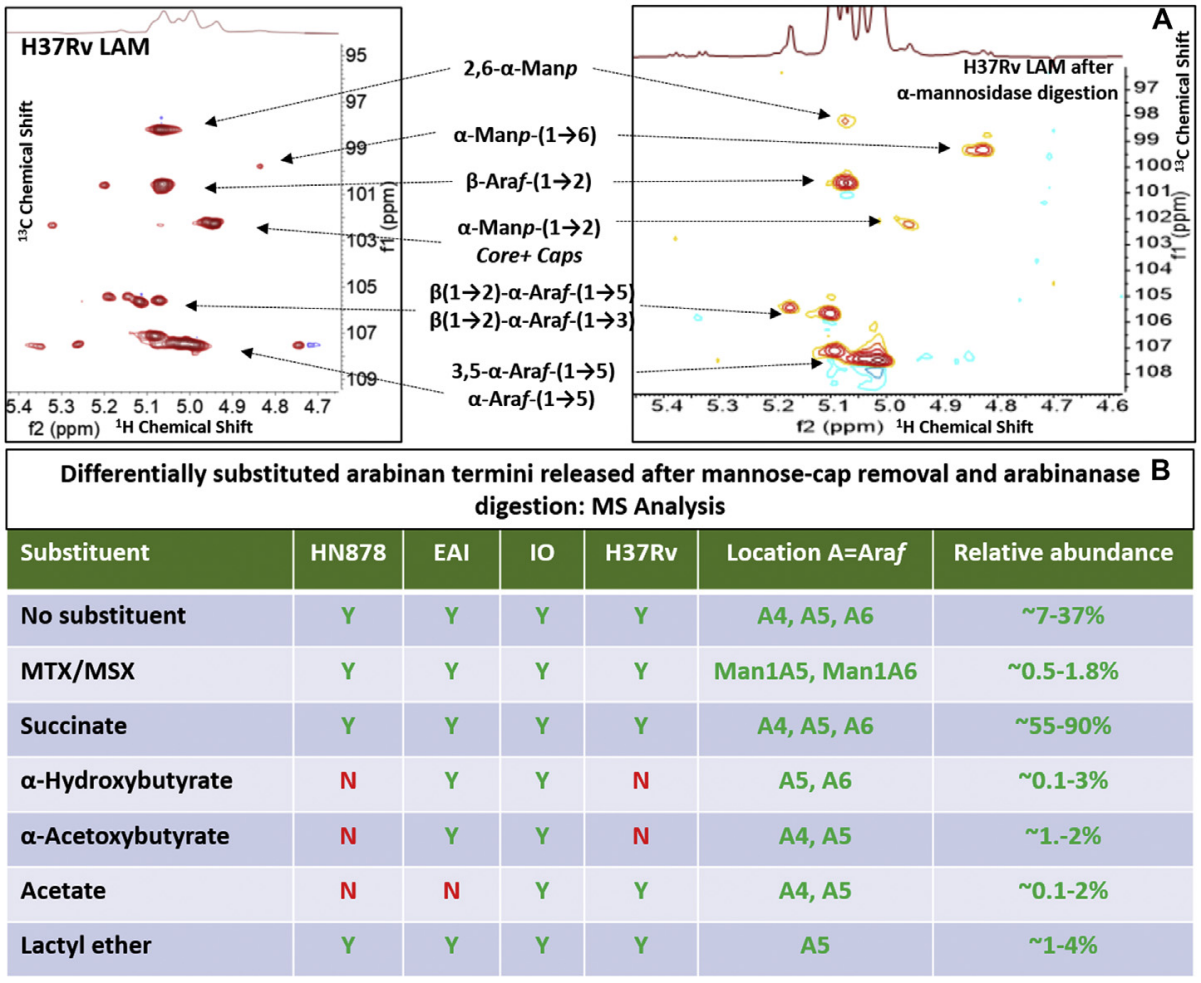
**Analyses of Arabian termini after enzyme digestions**. *A*, Changes in the glycosidic network of H37Rv LAM after α Mannosidase (Jack Bean). *B*, the normalized LC/MS abundances of differentially acylated arabinan termini (Ara_4_, Ara_5_, Ara_6_) released after sequential α-mannosidase (Mannose-cap removal) digestion followed by digestion with *endo*arabinanase. Percentages are based on ion intensity and are not absolute.

In order to unequivocally establish if the acyl substituents were on the terminal end Man*p* or not, LAM from each strain was first treated with α-mannosidase (Jack beans), and the residual glycan was passed through a Biogel P4 column. The ^1^H-NMR of digested LAM revealed that the acyl groups and MTX/MSX residues were still present in all 4× LAM molecules (Fig. S7, *A* and *B*). TOCSY experiment on the digested RvLAM sample, chosen as the pilot because of its available amount, confirmed the presence of succinates along with MTX/MSX. However, MTX/MSX cross peaks were of relatively low intensity. The possibility of slower nonspecific digestion of MTX-Man*p* linkages cannot be ruled out. Major changes were noticeable in the anomeric region of HSQC NMR spectrum (Fig. S7*C*). The two sets of overlapping protons for β-D-Ara*f*-(1→2)-Ara*f* arising from Man capped and uncapped arabinan termini merged into one peak, thereby confirming almost complete removal of mannose caps, as expected. A weak residual signal for t-α-D-Manp-(1→2)-Man*p* supports this observation. Unlike the parent RvLAM molecule, the peak for Man*p*-α-(1→6) appears more intense than 2,6-linked α-Man*p* (Fig. 9*A*). Although there is a collateral loss in structure, it does not deter any alteration in antibody recognition activity.

α-Mannosidase-treated LAM was subsequently digested with *endo*arabinanase. This allowed us to characterize the released arabinan termini (Ara_4_, Ara_5_, and Ara_6_) bearing acyl groups and MTX, without heterogeneity arising from mannose capping. We found that monosuccinylated Ara_4_ and Ara_5_ were major linear species with lesser amounts of branched Ara_6_ (Fig. 9*B*). Abundant nonacylated Ara_5_ and Ara_6_ were found in the RvLAM digest compared with the other LAM species in this study.

### Antigenic determinant in LAM

**Figure 10:**
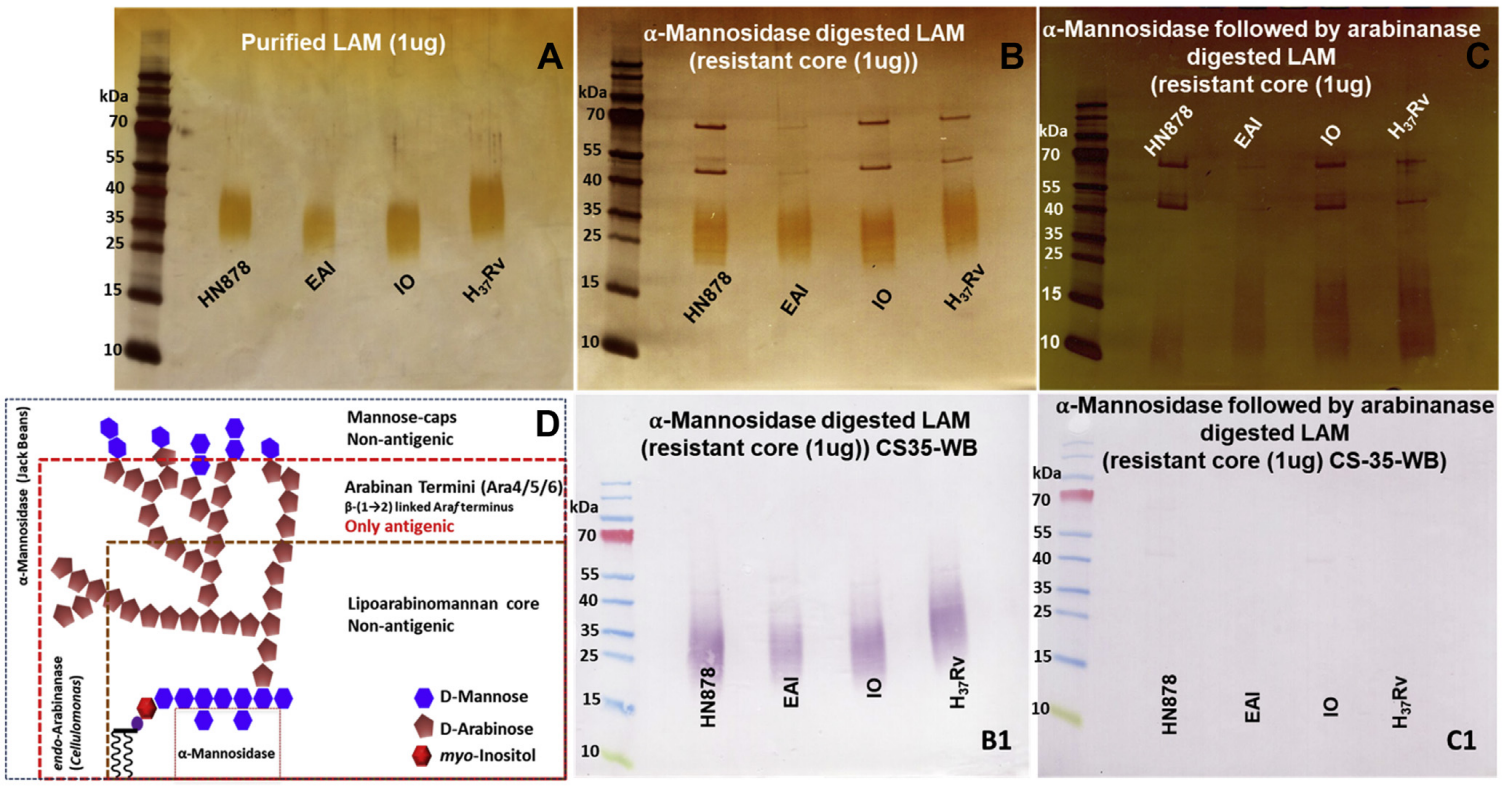
**The SDS-PAGE (Silver stain) and Western Blot (WB; mAb CS35 (Mouse) of four LAM samples and their enzyme resistant cores obtained after sequential digestion with α-mannosidase and endoarabinanase.***A*, SDS-PAGE of LAM (1 μg) electrophoretic mobility (35–40 kDa) of all clinical isolates. *B*, SDS-PAGE of α-mannosidase resistant core of LAM samples; clinical isolates have similar mobility (25–35 kDa) while RvLAM has reduced mobility (35–40 kDa): *B1*, WB of α-mannosidase treated LAM samples: all LAM samples preserved antigenicity towards mAb CS35. *C*, after digestion with *endo*arabinanase, the size spread of LAM shifted to 10 to 15 kDa. *C1*, the corresponding WB (mAb CS35) of arabinanase resistant LAM core: all LAM variants lost their binding capacity. *D*, summary of LAM antigenicity: mannose caps, arabinan core (mainly Ara*f*-α(1→5)), or mannan backbone is not responsible for binding to CS35. Only Ara_4_, Ara_5_, Ara_6_ specifically terminating with a Ara*f*-β(1→2) is contributing to mAb binding.

LAM extracted and purified from the Mtb strains were run on a 10–20% Tricine gel, followed by periodic acid-silver staining and transferred to nitrocellulose membrane for a Western Blot using mouse mAb CS35. The smear for RvLAM appeared to be running slower than the clinical isolates (Fig. 10*A*) indicating its larger size. As expected, CS35 mAb recognized LAM from each isolate equally well (Fig. 10*A*).

LAM, α-mannosidase-treated LAM, and *endo*arabinanase-treated enzyme resistant cores were analyzed by SDS/PAGE (Fig. 10*B*) followed by Western Blot using mAb CS35 (Fig. 10*B1*). As evident from the SDS/PAGE analyses, (Fig. 10*B*) there was only a shift of ∼2 kDa after mannosidase treatment and selective removal of Man capping did not abrogate any antibody binding activity. On the other hand, following the *endo*arabinanase treatment, which afforded only partial removal of the arabinan termini, the resistant core shifted down to 10–15 kDa (Fig. 10*C*) and lost all binding capacity to the antibody (Fig. 10*C1*). Compositional analyses of this material showed Ara:Man ratio of 0.9:1 indicating 57% of Ara still remaining and linkage analyses of this core by GC/MS showed some 2 Man*p*, 2 Ara*f* are present along with large amounts of 5-linked Ara*f* (Fig. S8, *A–C*).

### Docking studies

**Figure 11:**
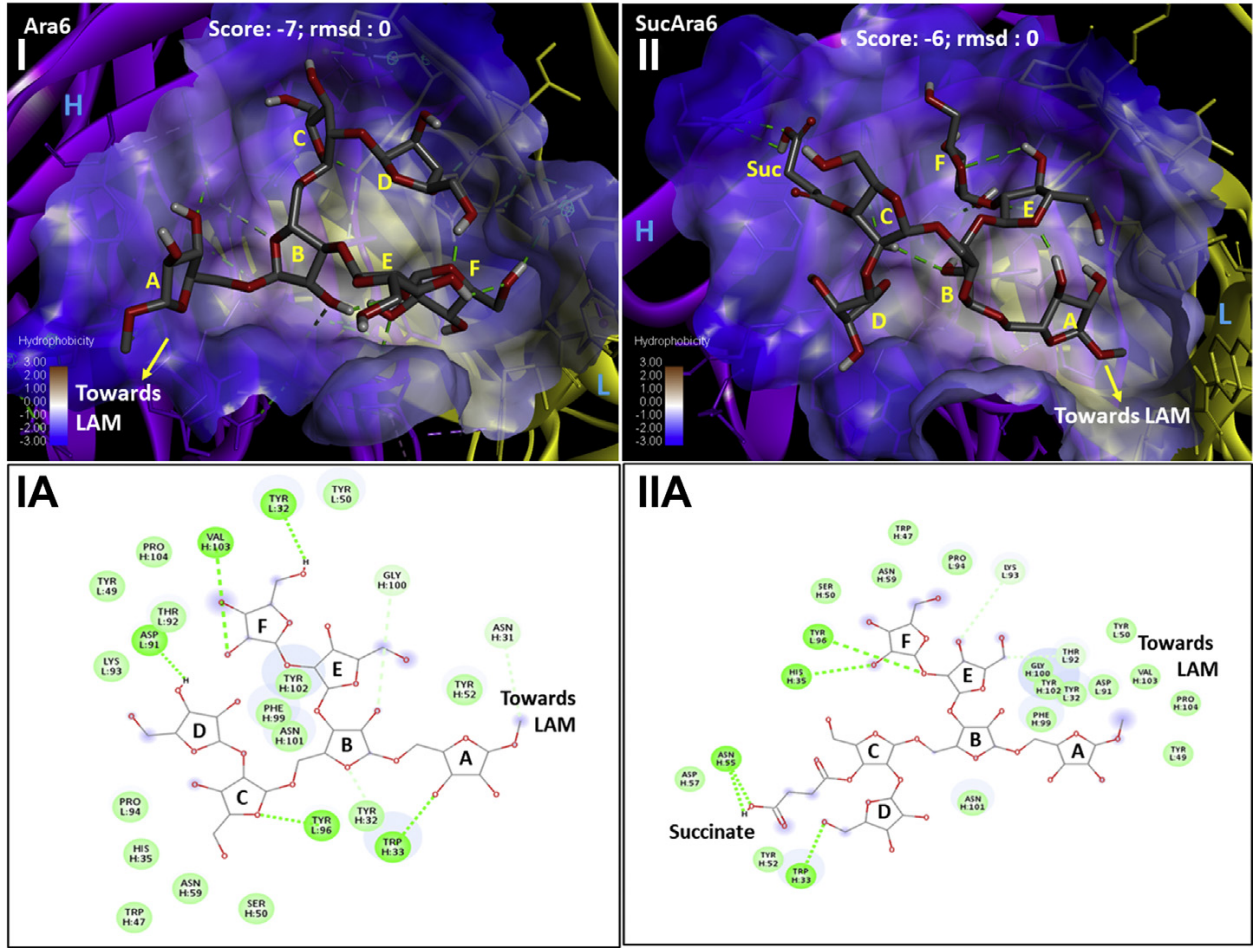
**Docking studies of Ara6 (*I* and *Ia*) and SucAra6 (*II* and *IIa*) with CS-35Fab (3HNS.pdb) on hydrophobic surface**: *Purple ribbon*, Heavy Chain and *Yellow ribbon*: Light Chain. Best pose (lowest score/binding energy (kCal/mol) and rmsd = 0) has been presented. Succinylation at the 3-position of ring-C-Ara*f* is unfavorable to binding compared to Ara6. Amino acid residues around Ara6 (*Ia*) were found to have similar orientation as reported (32). Upon succinylation, the binding conformation of the ligand (SucAra6) appeared (*II* and *IIa*) to be flipped with succinyl-residue posing outward. In the context of LAM-macromolecule, this may not be viable for binding.

The X-ray crystallographic investigation of synthetic methyl oligoarabinofuranosides (Ara_4_/Ara_6_)-CS35Fab recognition has been reported (32). The binding domain of CS35Fab and the conformational relevance of the nonreducing end of LAM (as represented by these synthetic substrates) are clearly understood. We attempted docking studies (PyRx-Vina (33) using hypothetical (MM2 energy minimized)(Ara_4_/Ara_6_) with or without a succinyl residue at the 3-position of the 2-linked-Ara*f* and CS35Fab. The lowest docking score (and root-mean-square deviation of atomic position; rmsd = 0) clearly showed that the binding of the succinylated Ara_4_/Ara_6_ to CS35-Fab is unfavorable in comparison to nonsuccinylated Ara_4_/Ara_6_ (Fig. 11 *I* and *Ia*). It is apparent (Discovery Studio visualizer) that the succinyl residue tends to pose outward the (Fig. 11 *II* and *IIa*) binding groove of CS35Fab indicating conformational mismatch (Figs. 11 and S9).

## Discussion

Current knowledge on the structure of LAM has resulted primarily from detailed studies on a few selected laboratory strains of M.tb (5, 34), *M. bovis bacillus* Calmette–Guérin (35), *M. smegmatis* (3), and *Mycobacterium kansasii* (22). Considerable effort has been invested in correlating particular structural features with aspects of the immunopathogenesis of TB (34). An outcome of these efforts is the consensus that the mannose-caps of ManLAM constitute the single most important structural entity engaged in phagocytosis by macrophages and subsequent events such as inhibition of phagosome/lysosome fusion and immunomodulation. Although most of the studies were based on LAM isolated from a culture, a promising start has been made with providing the description of LAM from urine of a TB positive patient and Mtb infected guinea pig/mouse lung only lately (21).

In our present study, we show that there is no marked difference among the LAM preparations from TB clinical isolates HN878, EAI, IO, and the laboratory reference strain H37Rv in their glycan structure. Notably the shared features are arabinan termini motifs, glycosidic linkages, and consistent substitution with succinates. In support, the ^1^H-^13^ C correlation spectra revealed that all 4xLAM samples have similar glycosidic linkages as reported for Mtb LAM (36, 37) pointing to the presence of distinct Ara_4_, Ara_5_, and Ara_6_ with and without Man capping.

The MTX/MSX substitution is common in all LAMs. MTX/MSX is a particular substituent ever since its discovery (26, 29) has been implicated in various functions such as regulatory (38), affinity to various antibodies, and as a contributor to a potent antigenicity (20). The distribution of this substitution is one/two per molecule of LAM. Therefore, in the context of antigen:(antibody)_n_ type recognition of LAM, MTX/MSX-termini may not have significant contribution toward affinity. For EAI-LAM, the terminal β-Ara*f* (t-Ara) appeared to be more abundant, whereas 2-linked Man*p* in lower abundance was observed by linkage analysis suggesting relatively lower mannose capping in EAI-LAM (Fig. S8*C*). Although succinylation is the most pronounced covalent modification of the LAM-arabinan, the TOCSY NMR of EAI-LAM (Fig. 3) revealed that there are other hitherto unreported acyl functions present in lesser abundance than the succinates, and mere presence of these features could dictate the antigenicity of LAM from these strains.

The MS analyses also supported the possibility of the presence of a lactate and acetate covalently linked in the nonreducing ends of LAM. In our present work, we exploited 2D NMR on LAM, MS and MS-MS analyses of enzyme released fragments and unambiguously established that these short chain carboxylic acids occupy the 3-OH position of the 2-linked Ara*f* (*i.e.*, penultimate Ara*f*). Only recently lactate and other short chain acids have been implicated to be present only as a contaminant in a LAM preparation (39). We conclusively show that these are covalently attached to the LAM-arabinan. Incidentally, the presence of lactate groups, associated with the arabinomannan domain of LAM, was also reported in *M.tb*, *M. smegmatis* and *M. leprae* (40).

α-Mannosidase-digested LAM was subsequently treated with *endo*arabinanase allowing us to characterize the released arabinan termini bearing acyl groups and MTX, without additional heterogeneity arising due to mannose capping. We found that succinyl residues mainly decorate the linear Ara_4_ and Ara_5_.

To date, our understanding of the biological significance of noncarbohydrate modifications of the arabinomannan remains speculative. To address this, we performed simple experiments and tested each variant against mAb CS35.

We set out to investigate the impact of structural variations in LAM onto the mAb binding. We hypothesized three scenarios of LAM in TB patients. The case of variability in mannose capping was addressed with α-mannosidase-digestion, the impact of the lack or presence of acylation was addressed with deacylation experiment, and the importance of structural arrangements in the arabinan termini was addressed by releasing the termini by digestion with *endo*arabinanase.

**Figure 12:**
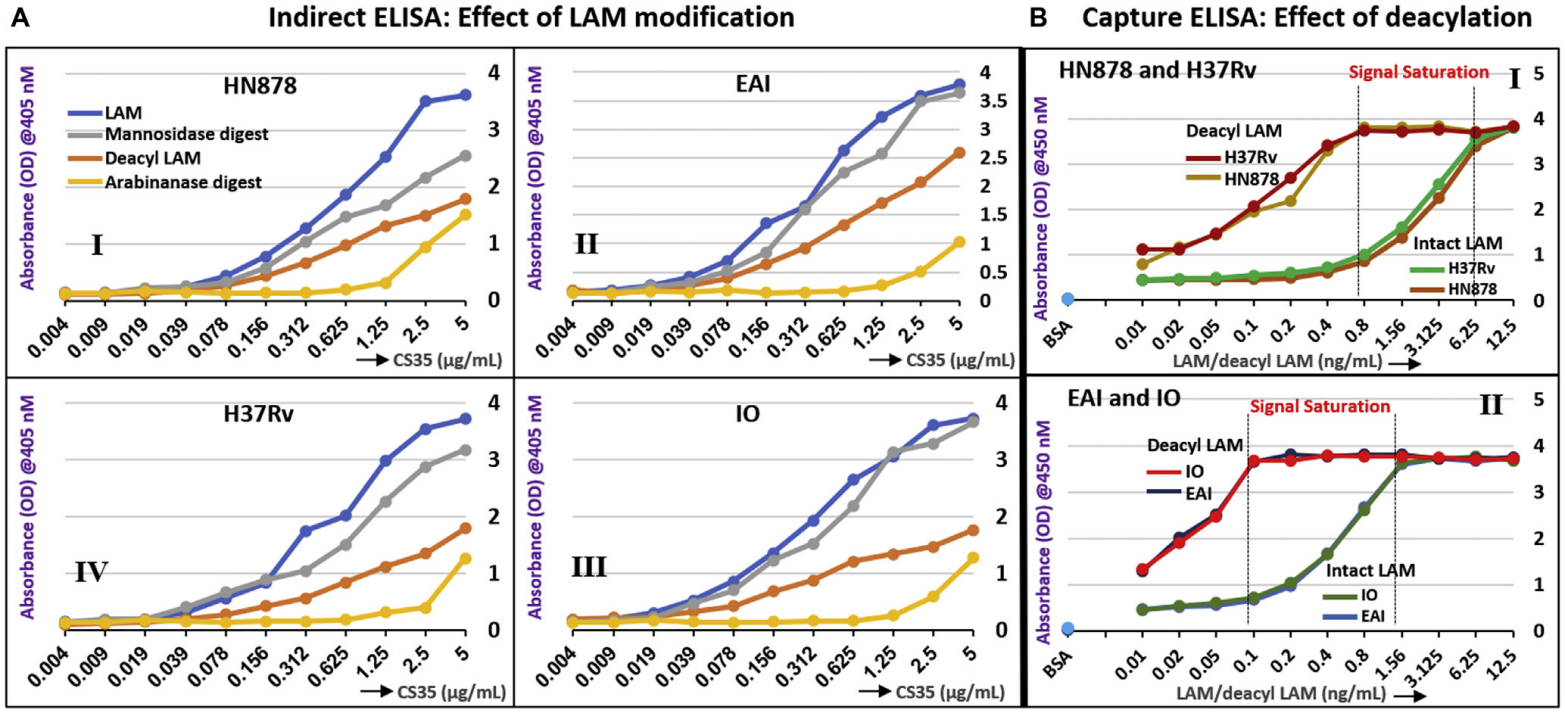
**Outcome of LAM modifications on mAb CS35 response by Indirect and Capture ELISA.***A*, indirect ELISA; *Blue line*: Intact LAM, *Gray line*: α-Mannosidase-treated LAM derivative, *Orange line*: deacylated (0.25 N NaOH treatment) LAM, *Yellow line*: Resistant LAM core after sequential digestion with α-mannosidase and *endo*arabinanase. *I*, HN878 LAM and derivatives, *II*, EAI LAM and derivatives, *III*, IO LAM and derivatives, *IV*, H37Rv LAM and derivatives. *B*, capture ELISA: CS35 (capture) and A194 (detection). Enhancement of ELISA sensitivity after deacylation. *I*, HN878 and H37Rv LAM and their deacylated LAM, *II*, EAI and IO LAM and their deacylated LAM.

We monitored the effect of Man cap removal by indirect ELISA whereby LAM/LAM derivatives were immobilized and subsequently detected by CS35, a well-known anti LAM murine mAb having strong binding affinity to LAM termini motifs. Earlier we reported (10) that CS35 is a broad-spectrum mAb with preferential affinity for Ara_6_. It also binds to the linear Ara_4_/Ara_5_ and to a lesser extent to the (Man_2_)_2_Ara_6_. Therefore, it is conceivable that the abundance of Ara_6_ should preferentially dictate the response to CS35 as LAM-binding epitopes. If, however, Ara_6_ is less abundant than (Man_2_)_2_Ara_6_, total mAb response may equate the sum of the responses from binding to all available Ara_6_ and (Man_2_)_2_Ara_6_. The LC/MS profile of all the four LAMs after digestion with *endo*arabinanase (Fig. 8*A*) revealed that the relative abundance of Ara_6_ in HN878, EAI, IO, and H37Rv is 6.7%, 16.1%, 11.3%, and 5.3%, respectively and that of (Man_2_)_2_Ara_6_ is 12.6%, 8.4%, 10.9%, and 16.3%, respectively. It is understandable that for EAI and IO LAM, the response from CS35 will have approximately 2-fold more contribution from Ara_6_ than for HN878 and H37Rv. This is what is supported by the ELISA (gray line; Fig. 12*A*, *I–IV*) as removal of Man caps does not influence the abundance of original Ara_6_. EAI and IO LAM did not show any drastic change in OD values after digestion with mannosidase (Fig. 12*A*, *II* and *III*). This justifies the reduced OD values for the mannosidase-digested HN878 and H37Rv LAM from their natives (blue line) by approximately 15–30%. These results indicate that the use of α-mannosidase as pretreatment reported for the detection of urinary LAM may be inconsistent (41). Ara_6_, Ara_4/5_, the major antigenic and abundant epitopes, have one common feature at the nonreducing end, *i.e.*, β-D-Ara*f*-(1→2)-α-D-Ara*f*-(1→5)-α-D-Ara*f*-(1→5)-. According to our findings, all the acyl groups as well as lactyl ether are at the 3-position of the 2-linked penultimate Ara*f*. Steric hindrance, offered by these acyls, may not allow the acylated epitopes to bind to CS35. The docking studies indicate that the binding of succinylated Ara_4_/Ara_6_ is less favored than their nonsuccinylated versions. Conformational mismatching may be responsible, thereby supporting our hypothesis. It is expected that deacylation, achieved by mild alkaline hydrolysis, should enable better availability of antigenic epitopes and consequently, enhancement of mAb-response and better sensitivity of immunoassay may be achieved (^1^H NMR of deacylated H37Rv and EAI-LAM are presented in Fig. S12). Our initial observation by indirect ELISA using 1 μg LAM (blue line)/deacyl LAM (orange line) and 2-fold concentration gradient of CS35 showed dramatic drop in OD values due to deacylation (Fig. 12*A*, *I–IV*). This was not unexpected because immobilization of LAM on polystyrene-ELISA plates relies on the PI anchor of LAM, and deacylation should remove these. Therefore, inefficient immobilization should lead to loss of antigen during washing and consequently, the mAb response should drop. To circumvent the antigen immobilization issue, the TB capture-ELISA (18) was employed. CS35 was used as the capture, and biotinylated A194-01 was used as the detection antibody while the LAM/deacyl-LAM concentration was varied with 2-fold dilution. Deacylated LAM from all the four strains reached signal saturation at 8–10-fold lower concentration than their native forms (Fig. 12*B*, *I* and *II*). These results demonstrate that the acyl groups impart some inhibitory effect towards intact LAM detection by capture ELISA, and deacylation is perhaps a rapid, cheap, and efficient way to achieve better sensitivity with the existing mAbs. However the caveat is, there is no evidence whether acyl functionalities such as mono-succinyl ester are present in LAM in biofluids.

Not unexpected, a drastic (∼70–80%) loss in CS35 response was observed with arabinanase resistant LAM-core compared with intact LAM (yellow line; Fig. 12*A*, *I–IV*) due to the loss of termini motifs.

## Conclusion

It is evident that the structure of LAM, from different demographic origin of *Mtb* species, varies. Our study centered on the contribution of nonreducing arabinan termini from a diagnostic perspective. The variation in relative abundance of non-mannose-capped, mannose-capped terminus, may not have a profound impact on immunoassay sensitivity if the mAb is preferentially specific to non-mannose-capped terminus like CS35. Different acyl groups found at the 3-position of the 2-linked Ara*f* of the antigenic epitopes partially inhibit Ab binding. Deacylation may prove to be advantageous as far as improving capture ELISA is concerned. Besides succinyl mono-ester, the presence of α-hydroxy/acetoxy butyryl ester in EAI and IO LAM is intriguing and needs further work to unveil biological implications. Importantly, when β-D-Ara*f*-(1→2)-α-D-Ara*f*-(1→5)-α-D-Ara*f*-(1→5)- arrangement is lost from the nonreducing terminus, antigenic response to CS35 was compromised pointing that overall, abundance of specific arrangement of arabinan termini is crucial for the LAM-mAb recognition.

The biological significance of the structural features and their variations among strains remains to be determined. That said, the finding of covalent substituents such as succinates, methylthioxylose (MTX), and other acyl functions modifying the glycan in all M.tb strains suggests that M.tb has evolved strategies as other prokaryotes to promote their survival in its host environments. From the diagnostic perspective, the importance of specific arabinan nonreducing-end motifs, irrespective of its modifications cannot be understated. These could be the ultimate feature requirement for TB specific Ab binding.

## Experimental procedures

### Preparation of M.tb cell biomass

Stocks of *M. tuberculosis* (*M.tb*) were originally received from the Trudeau mycobacterial culture collection (H37Rv), the laboratory of Dr James Musser (HN878), or the laboratory of Dr Sebastian Gagneux (T17 – “IO” and 91-0079 – “EAI”). All growth conditions were performed by incubation at 37 °C. Seed stocks were made by colony isolation, subculture to 7H9-OADC (100 ml), and freezing 0.8 ml samples in 20% glycerol. Genotyping was confirmed for the seed stocks by spoligotyping. Working stocks (3 × 1 ml) were used to inoculate 7H10-OADC plates with passage of bacterial lawns to 1 L Fernbach flasks containing GAS media (42); these cultures were twice passaged to achieve 16 L cultures of each strain. Cells were harvested, washed with water, weighed, and cell biomass ranging from 70 to 80 g was inactivated by gamma irradiation (Ce137 irradiation, 2.4 MRad, adsorbed dose). Cells were lyophilized and stored at –80 °C until use.

### Purification of Lipoarabinomannan

LAM was extracted from lyophilized cells as reported (32). Briefly, chloroform:methanol:water (10:10:3) extraction to remove polar surface lipids, cell lysis, and detergent partition to achieve LAM enriched fraction (36). Ethanol precipitation (–20 °C) produced LAM-enriched material. The precipitate was collected and resuspended at 50 mg/ml in endotoxin-free water and digested with proteinase K (0.1 mg/ml) to remove any residual proteins. After dialysis in running DI water (3500 MWCO cassette), the presence of LAM was confirmed by SDS-PAGE gel with periodic silver stain. Subsequently, tandem size-exclusion chromatography (Sephacryl-200 HR and Sephacryl-100 HR, 320 ml each) in LPS running buffer (0.2 M NaCl, 0.01 M Deoxycholic acid, 0.001 M EDTA in 0.01 M Tris, pH 8.0) was performed. Fractions (2.5 ml) were analyzed by SDS-PAGE. Pure LAM fractions were pooled and sequentially dialyzed (12,000 MWCO) against LPS dialysis buffer (minus deoxycholate), 1 M NaCl, running DI water, and endotoxin-free water. LAM was qualified by ^1^H- NMR. ImageJ (43, 44) was used to determine LAM concentration resolved on SDS-PAGE gel along with monosaccharide analysis.

### Monosaccharide composition

LAM samples were hydrolyzed with 2 M trifluoroacetic acid. The resulting monosaccharides were converted to alditol acetates and analyzed on a TSQ 8000 Evo triple-quad GC-MS (Thermo Scientific). The mass spectrum was scanned from *m/z* 50 to 500, data analysis was performed using Chromeleon Chromatography data system (Thermo Scientific). Quantitation was based on *3-O-methyl* -glucose, used as an internal standard (45).

### NMR experiments

All NMR spectra were recorded with ∼5 mg LAM in D_2_O (0.56 ml) at 25 °C. All PRESAT ^1^H NMR was recorded on a Bruker Neo 400 MHz NMR instrument. ^1^H-^13^ C correlation (HSQC) NMR spectra were recorded with 128 scans and relaxation delay 1.2 s on Bruker Neo 400 MHz NMR instrument. The default Bruker parameters were used for recording spectra. TOCSY (^1^H-^1^H correlation) NMR spectra were recorded with 256 scans on Varian Innova 500 MHz instrument. All chemical shifts are based on the reference to the HOD peak at 4.64 ppm. The default Varian parameters were used for recording spectra. Spectra were processed using MestReNova x64.

### α -mannosidase treatment conditions

LAM (1 mg/0.2 ml water) was treated with 0.085 ml (5 units) of α-Mannosidase (Jack Beans; Sigma; 3.5 mg/ml; pH 5.5) and incubated at 37 °C (water bath) for 20 h. The reaction mixture was then frozen at –80 °C for 2 h. After inactivation of the enzyme at 80 °C for 15 min, the reaction mixture was purified over Biogel-P4 column (30 cm × 1 cm, water).

### Digestion with endoarabinanase to release oligoarabinosides

LAM (100–200 μg) from each strain was digested with *endo*arabinanase as previously described (21) and the released arabinan fragments were separated from the resistant core by nanosep 3K Omega (Pall Corporation) and centrifuging at 14,000*g* for 20 min at room temp. An aliquot of samples was analyzed by SDS-PAGE to ensure digestion of LAM. The flow-through containing the released oligoarabinosides was analyzed by LC/MS.

### Immunoassays by Indirect and Capture ELISA

#### Indirect ELISA

Indirect ELISA was carried out as previously described (46). LAM samples (1 μg/ml) were taken in coating buffer (0.05 M sodium carbonate and sodium bicarbonate, pH 9.6) and applied to the 96-well plate (Corning, Costar) at 4 °C overnight. Wells were blocked by 1% BSA (Sigma Aldrich) in 1× PBS. Purified CS35 (5 μg/ml) was serially diluted 2-fold to derive a concentration curve and added to the wells and incubated at room temperature (90 min). The plates were then washed with 1× PBS containing 0.05% Tween-80 and incubated at room temperature (90 min) with the anti-mouse IgG alkaline phosphatase conjugated secondary antibody (1:2500 dilution). Subsequently, the alkaline phosphatase activity was measured (405 nm) after addition of p-nitrophenyl phosphate (pNPP) (Kirkegard and Perry Laboratories).

#### Capture ELISA

A 96-well polystyrene high binding microplate (Corning, Costar) was coated with CS35 (10 μg/ml in 1× PBS; 4 °C overnight). The plates were blocked (1% BSA in 1× PBS; 60 min). LAM and derivatives (in 1× PBS at the final concentration of 12.5 ng/ml) were serially diluted 2-fold and applied in duplicate to the plate/s. After incubation (90 min, RT) and wash, detection antibody (Biotinylated A194 hu mAb; 250 ng/ml) was applied. Streptavidin-Horseradish Peroxidase (R&D Systems) (25 min) followed by Ultra TMB ELISA chromogenic substrate (Thermo Scientific) were added to develop color. The reaction was stopped by adding 2 M sulfuric acid, and the optical density was measured at 450 nm.

#### Conditions for LC/MS

Enzyme-released oligosaccharides were subjected to ultraperformance liquid chromatography (UPLC) separation on a Waters Acquity UPLC H-Class system in line with a Bruker MaXis Plus quadrupole time-of-flight (QTOF) mass spectrometer (MS). Separation was performed in gradient mode with a Waters Atlantis T3 3.0 μm column (2.1 × 150 mm) at 40 °C. Mobile-phase components were 10 mM ammonium acetate in water (A) and 10 mM ammonium acetate in acetonitrile (B). The flow rate was 0.3 ml/min. The data were acquired in the negative electrospray ion (ESI) mode with a mass-to-charge ratio (*m/z*) range of 110−4000 at 1 Hz scan rate. For MS-MS experiments, the MS was in multiple reaction monitoring (MRM) scan mode with collision-induced dissociation (CID) energies of 40 eV and 60 eV on target masses with *m/z* width of 10 Da. Internal instrument mass-scale calibration was performed by infusing the Agilent ESI-L low-concentration tuning mix. Instrument controls were performed *via* the Bruker HyStar v4.1 software package. Data were processed using Bruker Compass 2.0 Data Analysis 4.4 software.

#### Docking studies

The methyl oligoarabinofuranosides with proper stereochemistry were drawn using Chemdraw 20.0 and 3D mm2 energy minimization was done. The protein structures of CS35Fab (3HNS and 3HNT) were obtained from Protein Data Bank. Docking studies were performed (3HNS with Ara_6_/SucAra_6_ and 3HNT with Ara_4_/SucAra_4_) on PyRx-Vina platform. The.pdbqt files were further analyzed using Discovery-studio-visualizer.

## Data availability

All data are contained within the manuscript, either in the main body or in the supporting information submitted with the manuscript

## Supporting information

This article contains supporting information.

## Conflict of interest

The authors declare that they have no conflicts of interest with the contents of this article.

## References

[1] Chatterjee D., Brennan P.J., Holst O., Brennan P.J., Itzstein v.M. (2009). Microbial Glycobiology: Structures, Relevance and Applications.

[2] Turner J., Torrelles J.B. (2018). Mannose-capped lipoarabinomannan in Mycobacterium tuberculosis pathogenesis. Pathog. Dis..

[3] Khoo K.-H., Dell A., Morris H.R., Brennan P.J., Chatterjee D. (1995). Inositol phosphate capping of the nonreducing termini of lipoarabinomannan from rapidly growing strains of *Mycobacterium*. J.Biol.Chem..

[4] Khoo K.H., Douglas E., Azadi P., Inamine J.M., Besra G.S., Mikusová K., Brennan P.J., Chatterjee D. (1996). Truncated structural variants of lipoarabinomannan in ethambutol drug-resistant strains of *Mycobacterium smegmatis* - inhibition of arabinan biosynthesis by ethambutol. J. Biol. Chem..

[5] Torrelles J.B., Sieling P.A., Zhang N., Keen M.A., McNeil M.R., Belisle J.T., Modlin R.L., Brennan P.J., Chatterjee D. (2012). Isolation of a distinct *Mycobacterium tuberculosis* mannose-capped lipoarabinomannan isoform responsible for recognition by CD1b-restricted T cells. Glycobiology.

[6] Guerardel Y., Maes E., Elass E., Leroy Y., Timmerman P., Besra G.S., Locht C., Strecker G., Kremer L. (2002). Structural study of lipomannan and lipoarabinomannan from *Mycobacterium chelonae*. Presence of unusual components with alpha 1,3-mannopyranose side chains. J. Biol. Chem..

[7] Briken V., Porcelli S.A., Besra G.S., Kremer L. (2004). Mycobacterial lipoarabinomannan and related lipoglycans: From biogenesis to modulation of the immune response. Mol. Microbiol..

[8] Appelmelk B.J., den Dunnen J., Driessen N.N., Ummels R., Pak M., Nigou J., Larrouy-Maumus G., Gurcha S.S., Movahedzadeh F., Geurtsen J., Brown E.J., Eysink Smeets M.M., Besra G.S., Willemsen P.T., Lowary T.L. (2008). The mannose cap of mycobacterial lipoarabinomannan does not dominate the Mycobacterium-host interaction. Cell Microbiol..

[9] Rivoire B., Ranchoff B., Chatterjee D., Gaylord H., Tsang A., Kolk A.H.J., Aspinall G.O., Brennan P.J. (1989). Generation of monoclonal antibodies to the specific sugar epitopes of *Mycobacterium avium* complex serovars. Infect. Immun..

[10] Amin A.G., De P., Spencer J.S., Brennan P.J., Daum J., Andre B.G., Joe M., Bai Y., Laurentius L., Porter M.D., Honnen W.J., Choudhary A., Lowary T.L., Pinter A., Chatterjee D. (2018). Detection of lipoarabinomannan in urine and serum of HIV-positive and HIV-negative TB suspects using an improved capture-enzyme linked immuno absorbent assay and gas chromatography/mass spectrometry. Tuberculosis (Edinb).

[11] Broger T., Sossen B., du Toit E., Kerkhoff A.D., Schutz C., Ivanova Reipold E., Ward A., Barr D.A., Mace A., Trollip A., Burton R., Ongarello S., Pinter A., Lowary T.L., Boehme C. (2019). Novel lipoarabinomannan point-of-care tuberculosis test for people with HIV: A diagnostic accuracy study. Lancet Infect. Dis..

[12] Choudhary A., Patel D., Honnen W., Lai Z., Prattipati R.S., Zheng R.B., Hsueh Y.C., Gennaro M.L., Lardizabal A., Restrepo B.I., Garcia-Viveros M., Joe M., Bai Y., Shen K., Sahloul K. (2018). Characterization of the antigenic heterogeneity of lipoarabinomannan, the major surface glycolipid of *Mycobacterium tuberculosis*, and complexity of antibody specificities toward this antigen. J. Immunol..

[13] Chatterjee D., Lowell K., Rivoire B., McNeil M., Brennan P.J. (1992). Lipoarabinomannan of *Mycobacterium tuberculosis*. Capping with mannosyl residues in some strains. J. Biol. Chem..

[14] Zhang A., Jumbe E., Krysiak R., Sidiki S., Kelley H.V., Chemey E.K., Kamba C., Mwapasa V., Garcia J.I., Norris A., Pan X.J., Evans C., Wang S.H., Kwiek J.J., Torrelles J.B. (2018). Low-cost diagnostic test for susceptible and drug-resistant tuberculosis in rural Malawi. Afr. J. Lab. Med..

[15] Lawn S.D., Kerkhoff A.D., Nicol M.P., Meintjes G. (2015). Underestimation of the true specificity of the urine lipoarabinomannan (LAM) point-of-care diagnostic assay for HIV-associated tuberculosis. J. Acquir. Immune Defic. Syndr..

[16] Shah M., Hanrahan C., Wang Z.Y., Dendukuri N., Lawn S.D., Denkinger C.M., Steingart K.R. (2016). Lateral flow urine lipoarabinomannan assay for detecting active tuberculosis in HIV-positive adults. Cochrane Database Syst. Rev..

[17] Paris L., Magni R., Zaidi F., Araujo R., Saini N., Harpole M., Coronel J., Kirwan D.E., Steinberg H., Gilman R.H., Petricoin E.F., Nisini R., Luchini A., Liotta L. (2017). Urine lipoarabinomannan glycan in HIV-negative patients with pulmonary tuberculosis correlates with disease severity. Sci. Transl. Med..

[18] Amin A.G., De P., Graham B., Calderon R.I., Franke M.F., Chatterjee D. (2021). Urine lipoarabinomannan in HIV uninfected, smear negative, symptomatic TB patients: Effective sample pretreatment for a sensitive immunoassay and mass spectrometry. Sci. Rep..

[19] Broger T., Tsionksy M., Mathew A., Lowary T.L., Pinter A., Plisova T., Bartlett D., Barbero S., Denkinger C.M., Moreau E., Katsuragi K., Kawasaki M., Nahid P., Sigal G.B. (2019). Sensitive electrochemiluminescence (ECL) immunoassays for detecting lipoarabinomannan (LAM) and ESAT-6 in urine and serum from tuberculosis patients. PLoS One.

[20] Sigal G.B., Pinter A., Lowary T.L., Kawasaki M., Li A., Mathew A., Tsionsky M., Zheng R.B., Plisova T., Shen K., Katsuragi K., Choudhary A., Honnen W.J., Nahid P., Denkinger C.M. (2018). A novel sensitive immunoassay targeting the 5-Methylthio-d-xylofuranose-lipoarabinomannan epitope meets the WHO's performance target for tuberculosis diagnosis. J. Clin. Microbiol..

[21] De P., Shi L., Boot C., Ordway D., McNeil M., Chatterjee D. (2020). Comparative structural study of terminal ends of lipoarabinomannan from mice infected lung tissues and urine of a tuberculosis positive patient. ACS Infect. Dis..

[22] Guerardel Y., Maes E., Briken V., Chirat F., Leroy Y., Locht C., Strecker G., Kremer L. (2003). Lipomannan and lipoarabinomannan from a clinical isolate of *Mycobacterium kansasi*i: Novel structural features and apoptosis-inducing properties. J. Biol. Chem..

[23] Coscolla M., Gagneux S. (2014). Consequences of genomic diversity in Mycobacterium tuberculosis. Semin. Immunol..

[24] Gagneux S., DeRiemer K., Van T., Kato-Maeda M., de Jong B.C., Narayanan S., Nicol M., Niemann S., Kremer K., Gutierrez M.C., Hilty M., Hopewell P.C., Small P.M. (2006). Variable host-pathogen compatibility in *Mycobacterium tuberculosis*. Proc. Natl. Acad. Sci. U. S. A..

[25] Hershberg R., Lipatov M., Small P.M., Sheffer H., Niemann S., Homolka S., Roach J.C., Kremer K., Petrov D.A., Feldman M.W., Gagneux S. (2008). High functional diversity in Mycobacterium tuberculosis driven by genetic drift and human demography. PLoS Biol..

[26] Turnbull W.B., Shimizu K.H., Chatterjee D., Homans S.W., Treumann A. (2004). Identification of the 5-methylthiopentosyl substituent in *Mycobacterium tuberculosis* lipoarabinomannan. Angew. Chem. Int. Ed. Engl..

[27] Bhamidi S., Scherman M.S., Rithner C.D., Prenni J.E., Chatterjee D., Khoo K.H., McNeil M.R. (2008). The identification and location of succinyl residues and the characterization of the interior Arabinan region allow for a model of the complete primary structure of Mycobacterium tuberculosis mycolyl arabinogalactan. J. Biol. Chem..

[28] Bialecki J.B., Axe U F., Attygale A.B. (2009). Hydroxycarbonyl anion (m/z 45), a diagnostic marker for alpha-hydroxy carboxylic acids. J. Mass. Spectrom..

[29] Treumann A., Xidong F., McDonnell L., Derrick P.J., Ashcroft A.E., Chatterjee D., Homans S.W. (2002). 5-Methylthiopentose: A new substituent on lipoarabinomannan in *Mycobacterium tuberculosis*. J. Mol. Biol..

[30] Carlsen L., Snyder J.P. (1978). Transition state:Sulfene and the CH2/SO2 potential energy surface. J. Org. Chem..

[31] Joe M., Sun D., Taha H., Completo G.C., Croudace J.E., Lammas D.A., Besra G.S., Lowary T.L. (2006). The 5-Deoxy-5-methylthio-xylofuranose residue in mycobacterial lipoarabinomannan. Absolute stereochemistry, linkage position, conformation, and immunomodulatory activity. J. Am. Chem. Soc..

[32] Murase T., Zheng R.B., Joe M., Bai Y., Marcus S.L., Lowary T.L., Ng K.K. (2009). Structural insights into antibody recognition of mycobacterial polysaccharides. J. Mol. Biol..

[33] Trott O., Olson J. (2010). Autodoc Vina: Improving the speed and accuracy of docking with new scoring function, efficient optimization and multithreading. J. Comput. Chem.

[34] Kaur D., Obregon-Henao A., Pham H., Chatterjee D., Brennan P.J., Jackson M. (2008). Lipoarabinomannan of Mycobacterium: Mannose capping by a multifunctional terminal mannosyltransferase. Proc. Natl. Acad. Sci. U. S. A..

[35] Prinzis S., Chatterjee D., Brennan P.J. (1993). Structure and antigenicity of lipoarabinomannan from *Mycobacterium bovis* BCG. J. Gen. Microbiol..

[36] Torrelles J.B., Khoo K.H., Sieling P.A., Modlin R.L., Zhang N., Marques A.M., Treumann A., Rithner C.D., Brennan P.J., Chatterjee D. (2004). Truncated structural variants of lipoarabinomannan in *Mycobacterium leprae* and an ethambutol-resistant strain of *Mycobacterium tuberculosis*. J. Biol. Chem..

[37] Torrelles J.B., Knaup R., Kolareth A., Slepushkina T., Kaufman T.M., Kang P.B., Hill P., Brennan P.J., Chatterjee D., Belisle J.T., Musser J.M., Schlesinger L.S. (2008). Identification of mycobacterium tuberculosis clinical isolates with altered phagocytosis by human macrophages due to a truncated lipoarabinomannan. J. Biol. Chem..

[38] Angala S.K., McNeil M.R., Shi L., Joe M., Pham H., Zuberogoitia S., Nigou J., Boot C.M., Lowary T.L., Gilleron M., Jackson M. (2017). Biosynthesis of the methylthioxylose capping motif of lipoarabinomannan in Mycobacterium tuberculosis. ACS Chem. Biol..

[39] Angala S.K., Palcekova Z., Belardinelli J.M., Jackson M. (2018). Covalent modifications of polysaccharides in mycobacteria. Nat. Chem. Biol..

[40] Hunter S.W., Gaylord H., Brennan P.J. (1986). Structure and antigenicity of the phosphorylated lipopolysaccharide antigens from the leprosy and tubercle bacilli. J. Biol. Chem..

[41] Garcia J.I., Melendez J., Alvarez R., Mejia-Chew C., Kelley H.V., Sidiki S., Castillo A., Mazariegos C., Lopez-Tellez C., Forno D., Ayala N., Balada-Llasat J.M., Mejia-Villatoro C.R., Wang S.H., Torrelles J.B. (2020). Accuracy of the tuberculosis point-of-care Alere determine lipoarabinomannan antigen diagnostic test using alpha-mannosidase treated and untreated urine in a cohort of people living with HIV in Guatemala. AIDS Res. Ther..

[42] Takayama K., Schnoes H.K., Armstrong E.L., Boyle W.R. (1975). Site of inhibitory action of isoniazid in the synthesis of mycolic acids in *Mycobacterium smegmatis*. J. Lip. Res..

[43] Abramoff M.D., Magalhaes P.J., Ram S.J. (2004). Image processing with ImageJ. Biophotonics Int..

[44] Schneider C.A., Rasband W.S., Eliceiri K.W. (2012). NIH image to ImageJ: 25 years of image analysis. Nat. Methods.

[45] McNeil M., Chatterjee D., Hunter S.W., Brennan P.J., Ginsberg V. (1989). Methods Enzymol.

[46] Britton W.J., Hellqvist L., Basten A., Raison R.L. (1985). Mycobacterium leprae antigens involved in human immune responses. I. Identification of four antigens by monoclonal antibodies. J. Immunol..

