# Supplementary material for: Structural implications of lipoarabinomannan glycans from global clinical isolates in diagnosis of Mycobacterium tuberculosis infection
Source: J Biol Chem. 2021 Sep 30;297(5):101265. doi: 10.1016/j.jbc.2021.101265 (PMC8531672; doi:10.1016/j.jbc.2021.101265)
Supplement: Supplementary file 1 — Supporting information [file mmc1.docx]

**Supporting Information**

**Structural Implications of Lipoarabinomannan Glycans from Global Clinical Isolates in Diagnosis of *Mycobacterium tuberculosis* Infection**

Prithwiraj De^a^, Anita G. Amin^a^, Danara Flores^a^, Anne Simpson^a^, Karen Dobos^a*^, and Delphi Chatterjee^a*^

^a^ Mycobacteria Research Laboratories, Department of Microbiology, Immunology and Pathology

Colorado State University, Fort Collins, CO 80523, USA.

* Corresponding authors E.Mail: [delphi.chatterjee@colostate.edu](mailto:delphi.chatterjee@colostate.edu); Tel: +1 970 491-7495

[Karen.Dobos@colostate.edu](mailto:Karen.Dobos@colostate.edu); Tel: +1 970 491-2229

CoAuthors E.Mail:

[prithwiraj.de@colostate.edu](mailto:prithwiraj.de@colostate.edu)

[anita.amin@colostate.edu](mailto:anita.amin@colostate.edu)

[danara.flores@rams](mailto:danara.flores@rams) colostate.edu

[anne.simpson@colostate.edu](mailto:anne.simpson@colostate.edu)

**Running Title:** *Lipoarabinomannan from global tuberculosis clinical isolates*

**Contents**

**Fig S1:** ^1^H NMR (watersup, D_2_O, 128 scans; 400 MHz) spectra of IO-LAM (Top), EAI-LAM (Middle) and HN878-LAM (Bottom). **Page S3**

**Fig S2A:** ^1^H-^13^C Correlation (HSQC, D_2_O, 128 scans, 400 MHz) NMR spectrum of EAI-LAM. **Page S3**

**Fig S2B:** ^1^H-^13^C Correlation (HSQC, D_2_O, 128 scans, 400 MHz) NMR spectrum of IO-LAM. **Page S4**

**Fig S2C:** ^1^H-^13^C Correlation (HSQC, D_2_O, 128 scans, 400 MHz) NMR spectrum of HN878-LAM. **Page S4**

**Fig S2D:** ^1^H-^13^C Correlation (HSQC, D_2_O, 128 scans, 400 MHz) NMR spectrum of H37Rv-LAM. **Page S5**

**Fig S3A:** ^1^H-^1^H Correlation (TOCSY, D_2_O, 256 scans, 500 MHz) NMR spectrum of HN878-LAM. **Page S5**

**Fig S3B:** ^1^H-^1^H Correlation (TOCSY, D_2_O, 256 scans, 500 MHz) NMR spectrum of IO-LAM. **Page S6**

**Fig S3C:** ^1^H-^1^H Correlation (TOCSY, D_2_O, 256 scans, 500 MHz) NMR spectrum of H37Rv-LAM. **Page S6**

**Fig S4: The extracted ion chromatograms (EIC, LC-MS, negative-ion) of monosuccinylated Man_2_Ara_5_ (*m/z* 1101.34 [M-H]^-1^) for HN878, EAI and IO LAM and MS/MS analysis (negative ion, 40 ev, HN878-LAM). Page S7**

**Fig S5A:** **LC/MS-MS Fragmentation of (α-acetoxy butyryl Ara4; m/z 673 [M-H]¯; EAI LAM) (40 eV, negative ion, ESI-Collision-induced dissociation) obtained after sequential enzymatic digestions. Page S7**

**Fig S5B:** The Chromatograms of the explained ions for 2-Acetoxybutyryl Ara_4_. **Page S8**

**Fig S6:** Extracted ion chromatogram (from LC/MS, negative ion) of MSXMan1Ara5 (m/z 1017.29 [M-H]^-^) and MSX MSXMan1Ara6 (m/z 1149.33 [M-H]^-^) for EAI-, IO- and HN878-LAM after one-step arabinanase digestion. **Page S8**

**Fig S7A:** ^1^H-^1^H Correlation (TOCSY, D_2_O, 256 scans, 500 MHz) NMR spectrum of H37Rv-LAM after α-Mannosidase digestion. **Page S9**

**Fig S7B:** ^1^H NMR (watersupp, D_2_O, 128 scans, 400 MHz) of EAI-LAM after α-Mannosidase digestion (Bottom); a comparison with intact-EAI-LAM (Top). **Page S9**

**Fig S7C:** ^1^H-^13^C Correlation (HSQC, D_2_O, 128 scans, 400 MHz) NMR spectrum of H37Rv-LAM after α-Mannosidase digestion. **Page S10**

**Fig S8A:** Monosaccharide-Alditol acetate (GC/MS chromatogram, TIC) assay of Mannosidase digested H37Rv-LAM (Top), Intact-H37Rv-LAM (Middle) and Mannosidase-Arabinanase-digested H37Rv-LAM (Bottom). **Page S11**

**Fig S8B:** Glycosidic-linkage (Permethylation-Alditol acetate) (GC/MS chtomatogram, TIC) analysis of Mannosidase-Arabinanase-digested H37Rv-LAM (Top), Mannosidase digested H37Rv-LAM (Middle) and Intact-H37Rv-LAM (Bottom). **Page S11**

**Fig S8C:** Relative percentage values of major glycosidic linkages of LAMs from clinical isolates. **Page S12**

**Fig S9: Docking studies of Ara4 and SucAra4 with CS-35Fab (3HNT.pdb) on hydrophobic surface. page S12**

**Fig S10:** ^1^H NMR (Watersupp, D_2_O, 256 Scans, 400 MHz) of deacylated (0.25 N Aq. NaOH; 37°C, 14 h) H37Rv-(Top) and EAI-LAM (Bottom). **Page S13**

**Materials and Methods: Page S13-S15**


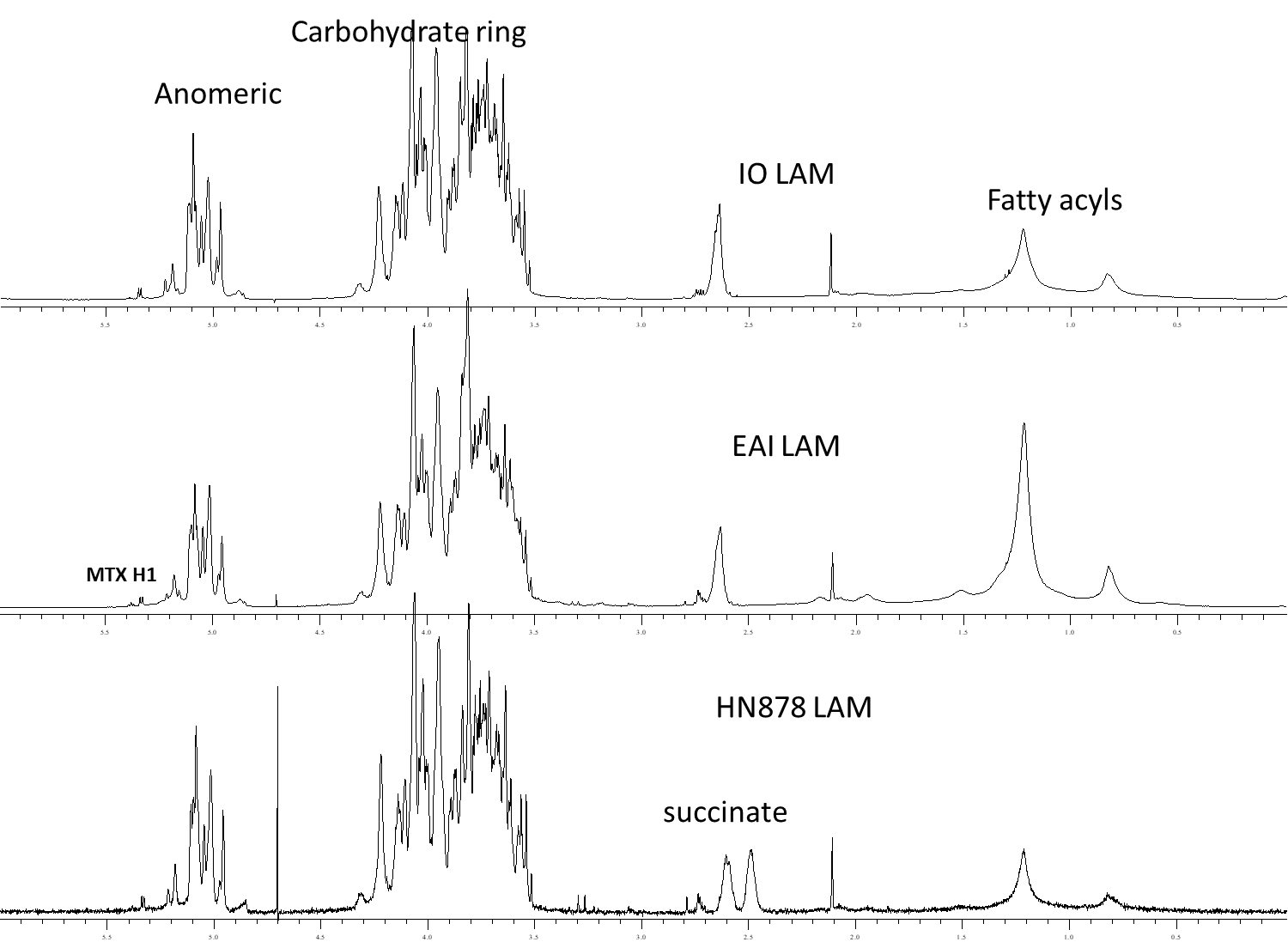


**Fig S1:** ^1^H NMR (watersupp, D_2_O, 128 scans; 400 MHz) spectra of IO-LAM (Top), EAI-LAM (Middle) and HN878-LAM (Bottom).


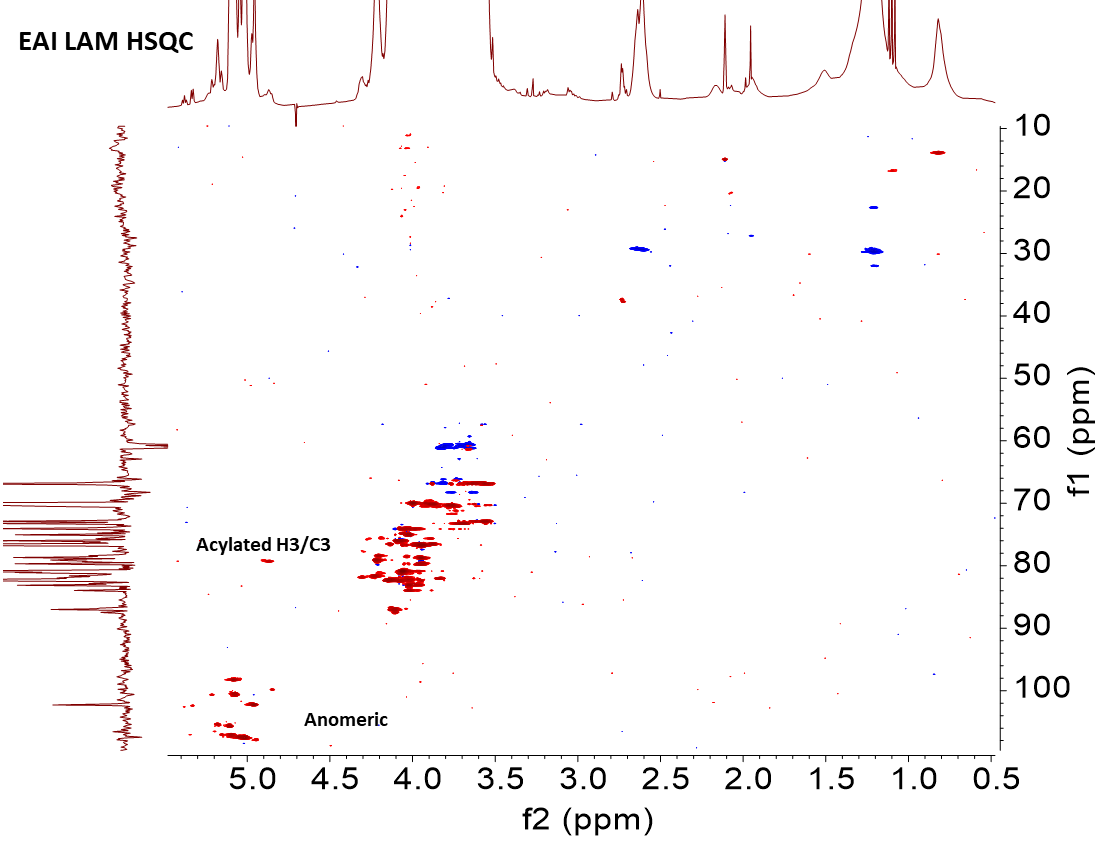


**Fig S2A:** ^1^H-^13^C Correlation (HSQC, D_2_O, 128 scans, 400 MHz) NMR spectrum of EAI-LAM.


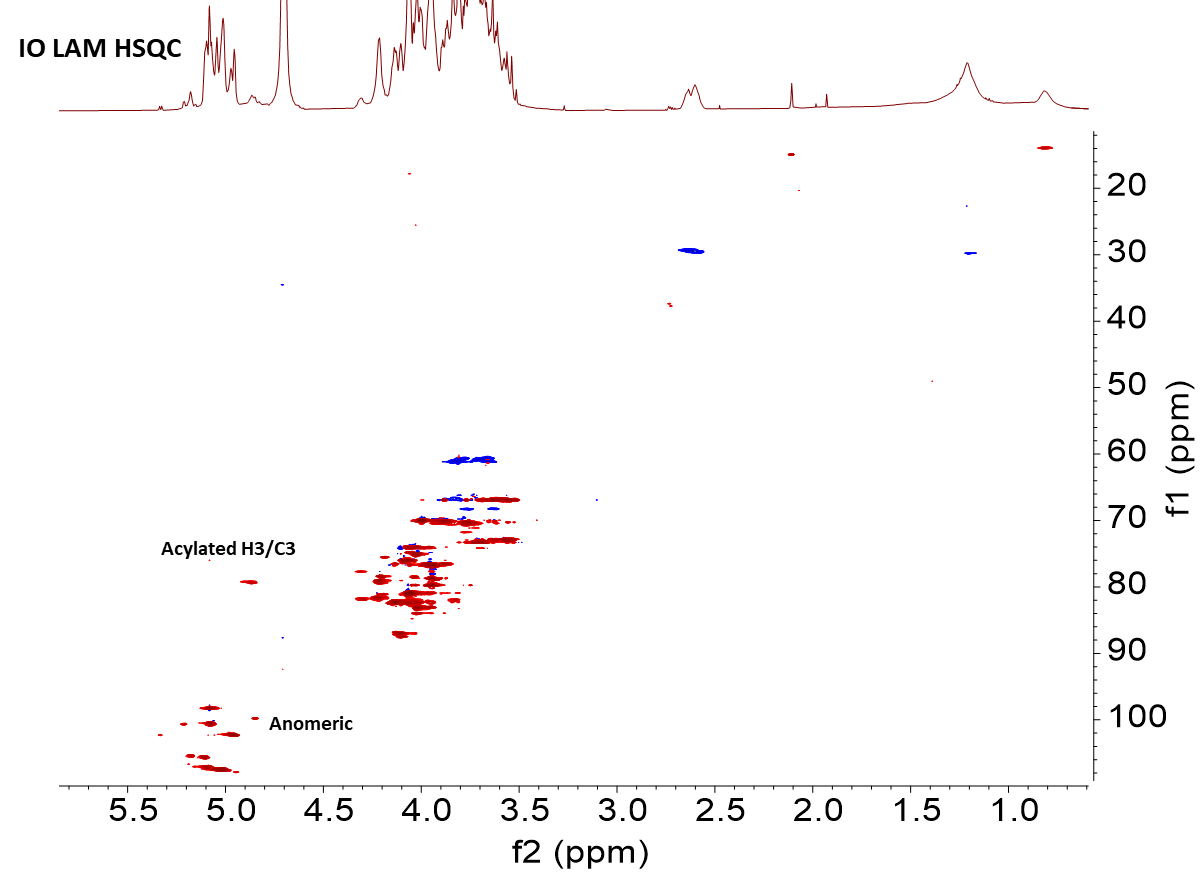


**Fig S2B:** ^1^H-^13^C Correlation (HSQC, D_2_O, 128 scans, 400 MHz) NMR spectrum of IO-LAM.


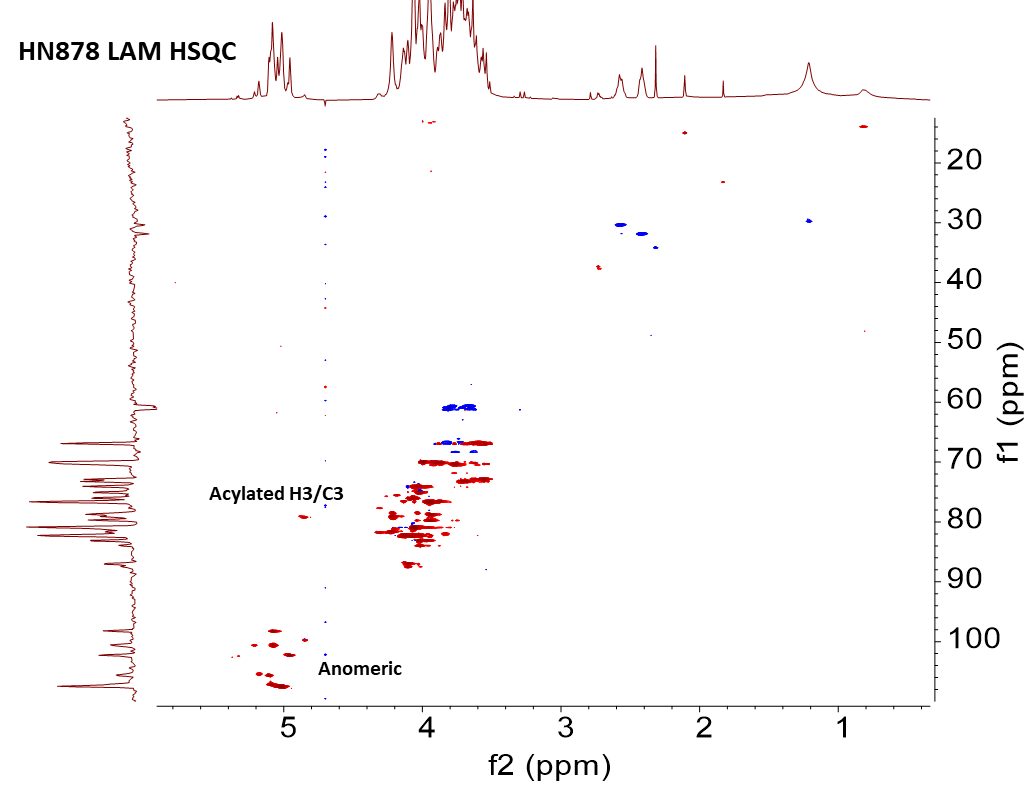


**Fig S2C:** ^1^H-^13^C Correlation (HSQC, D_2_O, 128 scans, 400 MHz) NMR spectrum of HN878-LAM.


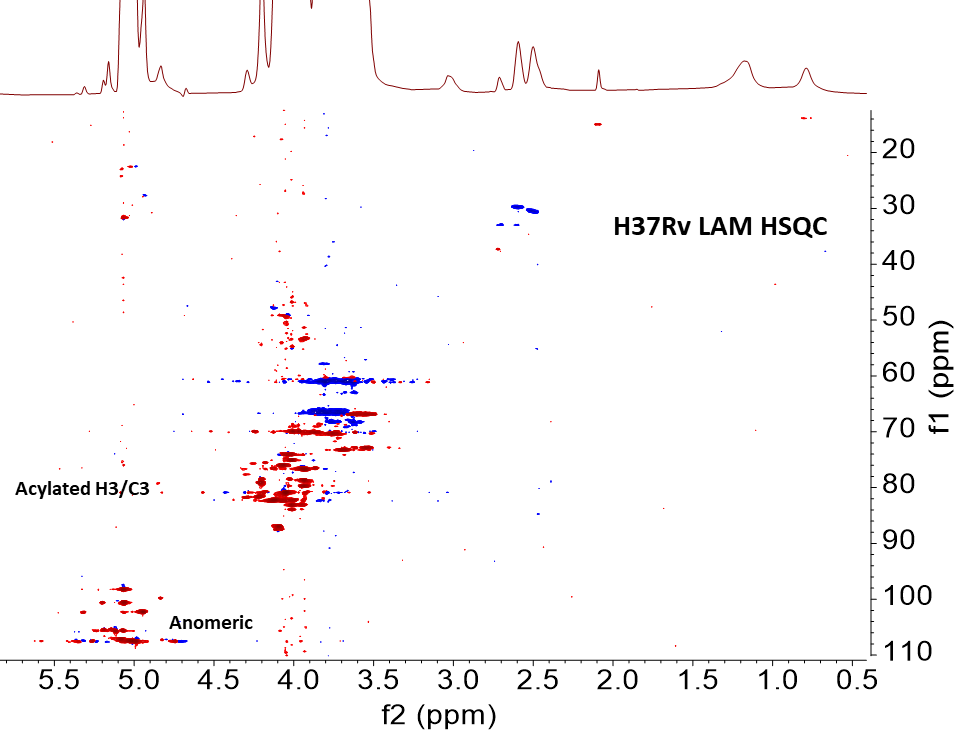


**Fig S2D:** ^1^H-^13^C Correlation (HSQC, D_2_O, 128 scans, 400 MHz) NMR spectrum of H37Rv-LAM.


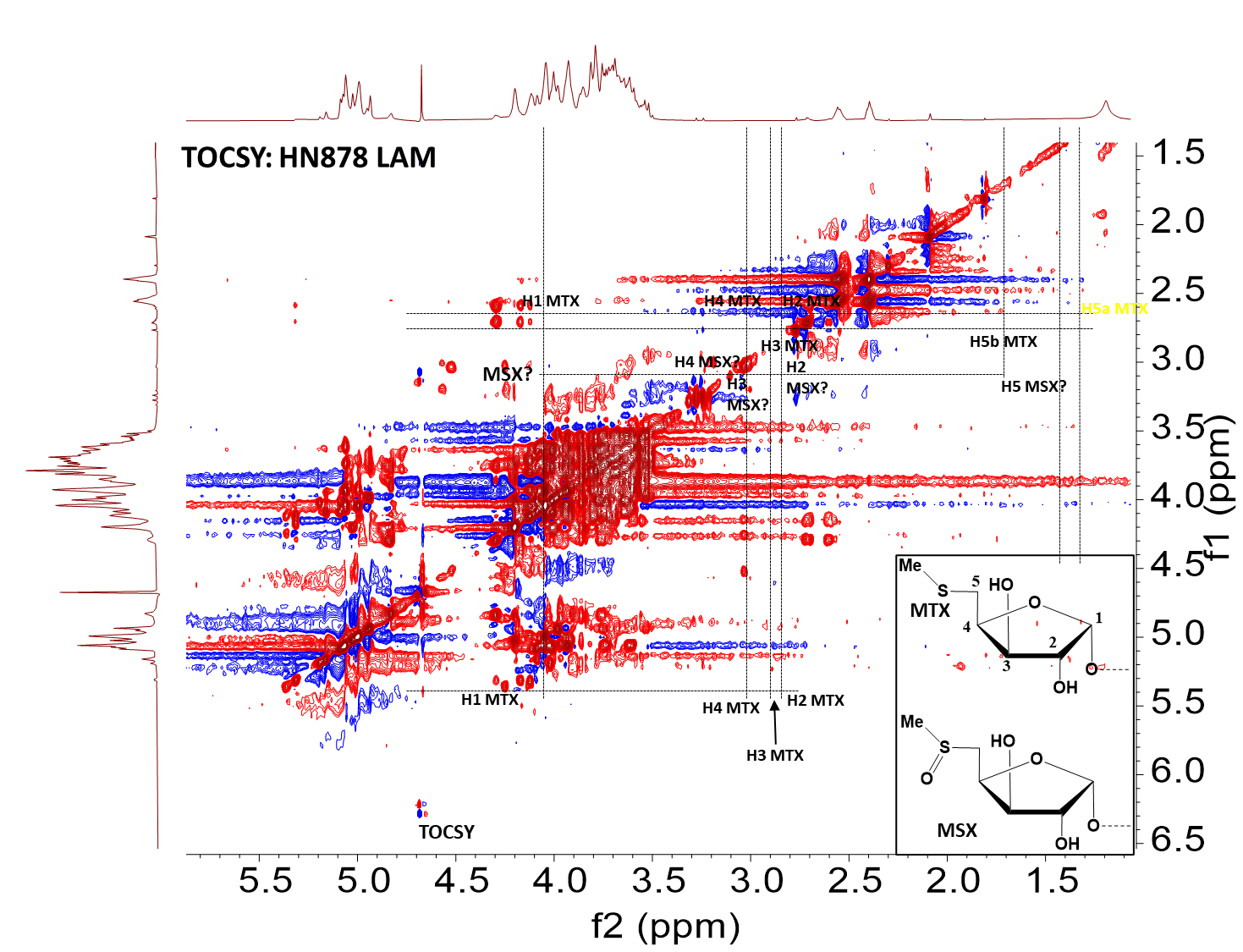


**Fig S4A:** ^1^H-^1^H Correlation (TOCSY, D_2_O, 256 scans, 500 MHz) NMR spectrum of HN878-LAM.


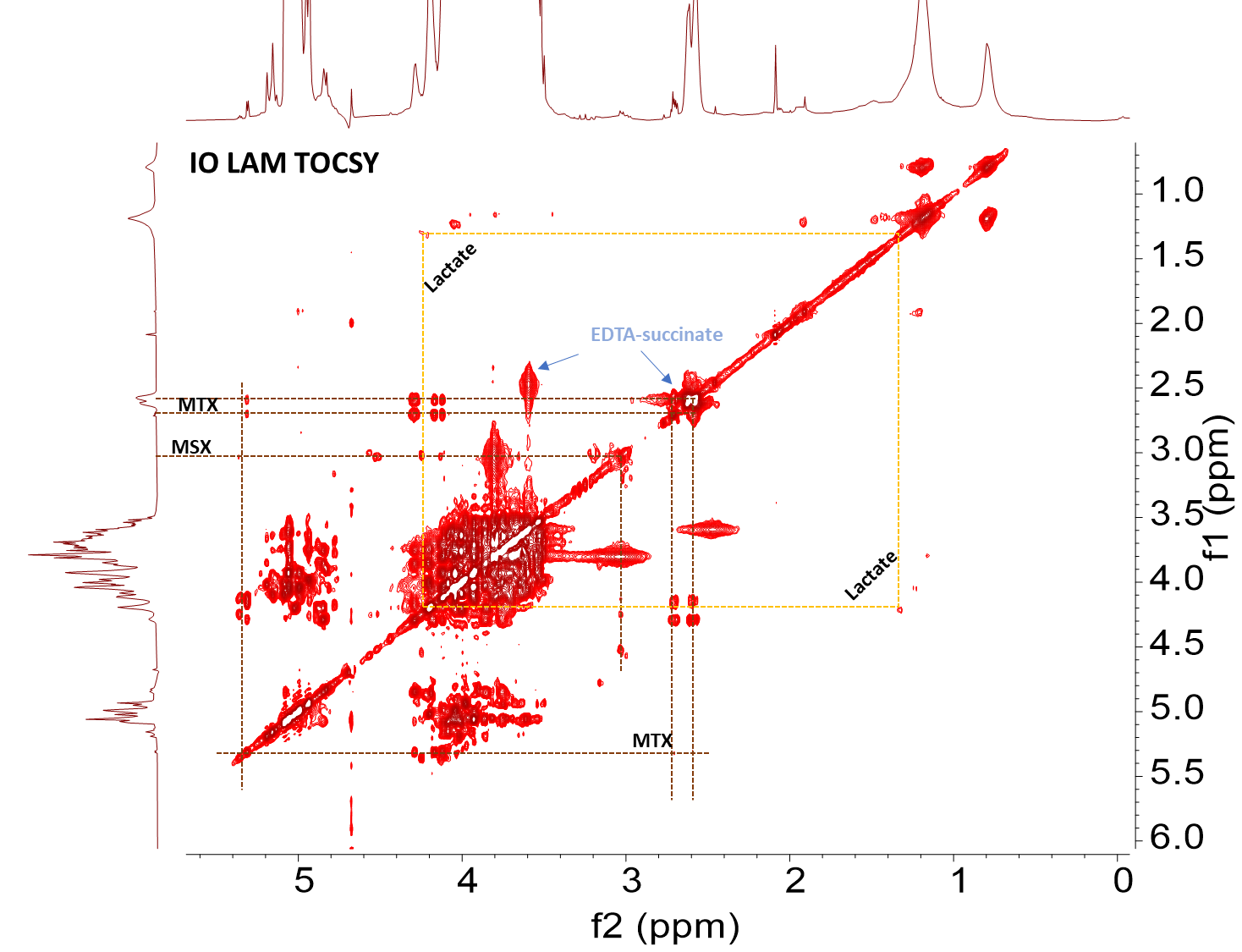


**Fig S4B:** ^1^H-^1^H Correlation (TOCSY, D_2_O, 256 scans, 500 MHz) NMR spectrum of IO-LAM.


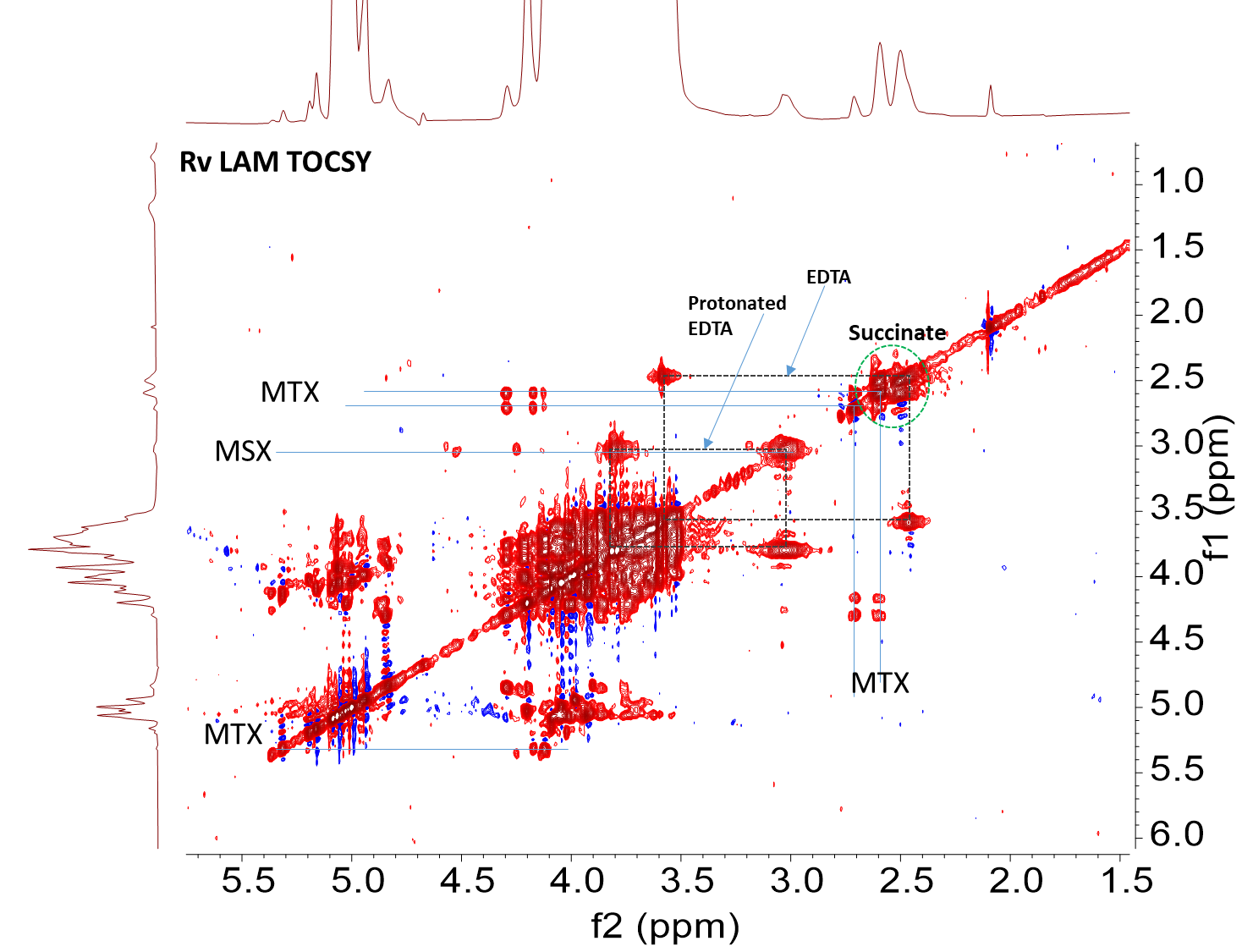


**Fig S4C:** ^1^H-^1^H Correlation (TOCSY, D_2_O, 256 scans, 500 MHz) NMR spectrum of H37Rv-LAM.


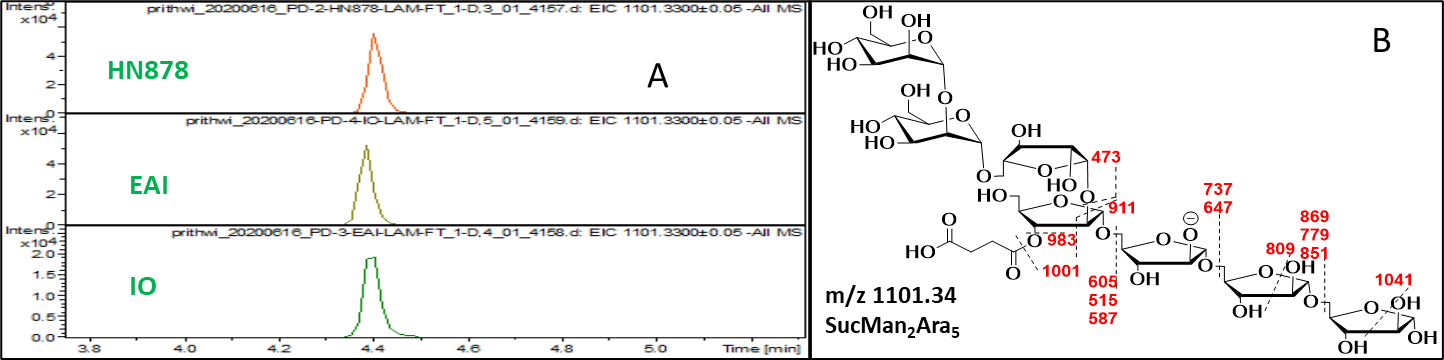


**Fig S4:** **The extracted ion chromatograms (EIC, LC-MS, negative-ion) of monosuccinylated Man_2_Ara_5_ (*m/z* 1101.34 [M-H]^-1^) for HN878, EAI and IO LAM**. These oligosaccharides were obtained after *endo*arabinanase digestion (released arabinan termini, <3 kDa) of LAM. **E.** The analyzed tandem-MS fragmentation (negative ion, CE 40 eV) of m/z 1101.34 [M-H]¯. The analysis suggests the location of succinate at the H-3 of a 2-linked arabinose, a linear arrangement of 5-Ara*f*-residues in Ara_5_ similar to our previous report (21).


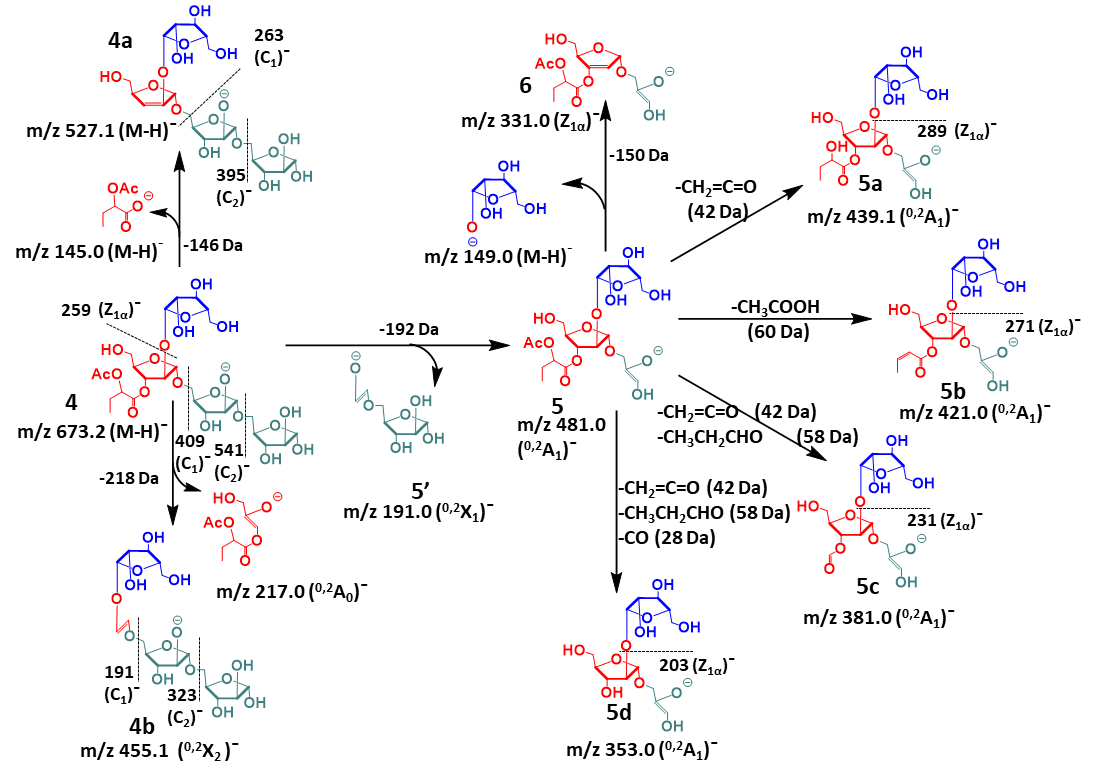


**Fig S5A:** **LC/MS-MS Fragmentation of (α-acetoxy butyryl Ara4; m/z 673 [M-H]¯; EAI LAM) (40 eV, negative ion, ESI-Collision-induced dissociation) obtained after sequential enzymatic digestions.**

**
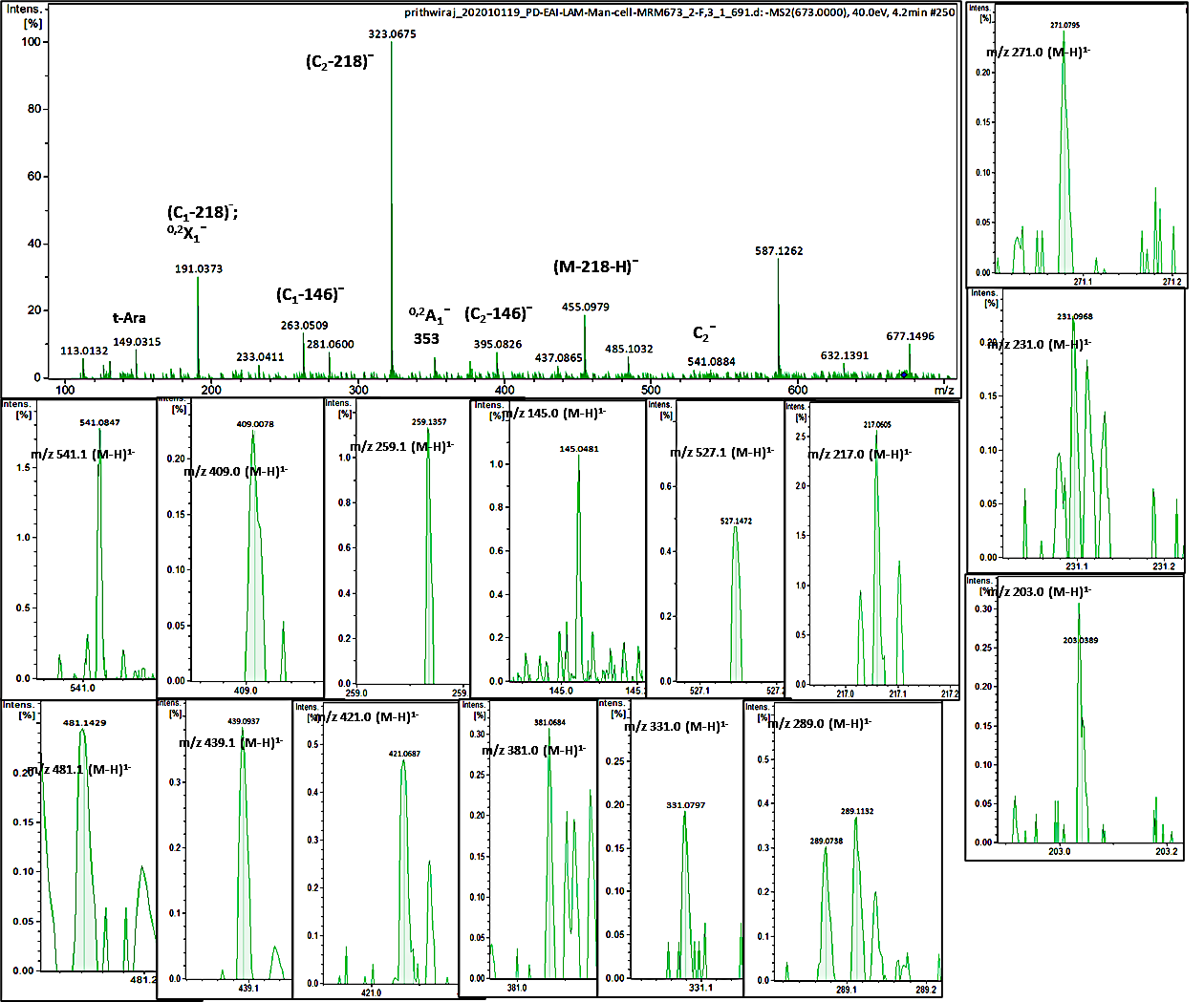
**

**Fig S5B:** The Chromatograms of the explained ions for 2-Acetoxybutyryl Ara_4_.


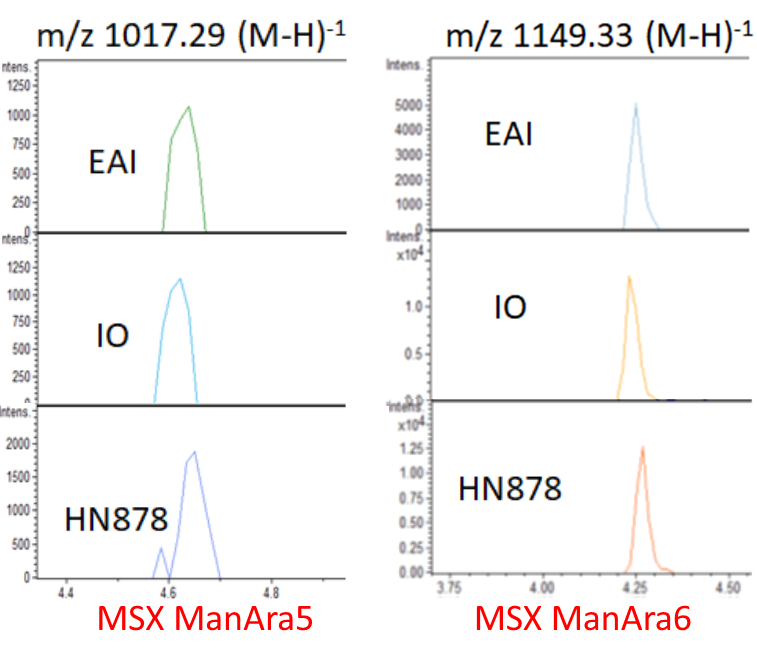


**Fig S6:** Extracted ion chromatogram (from LC/MS, negative ion) of MSXMan1Ara5 (m/z 1017.29 [M-H]^-^) and MSX MSXMan1Ara6 (m/z 1149.33 [M-H]^-^) for EAI-, IO- and HN878-LAM after one-step arabinanase digestion.


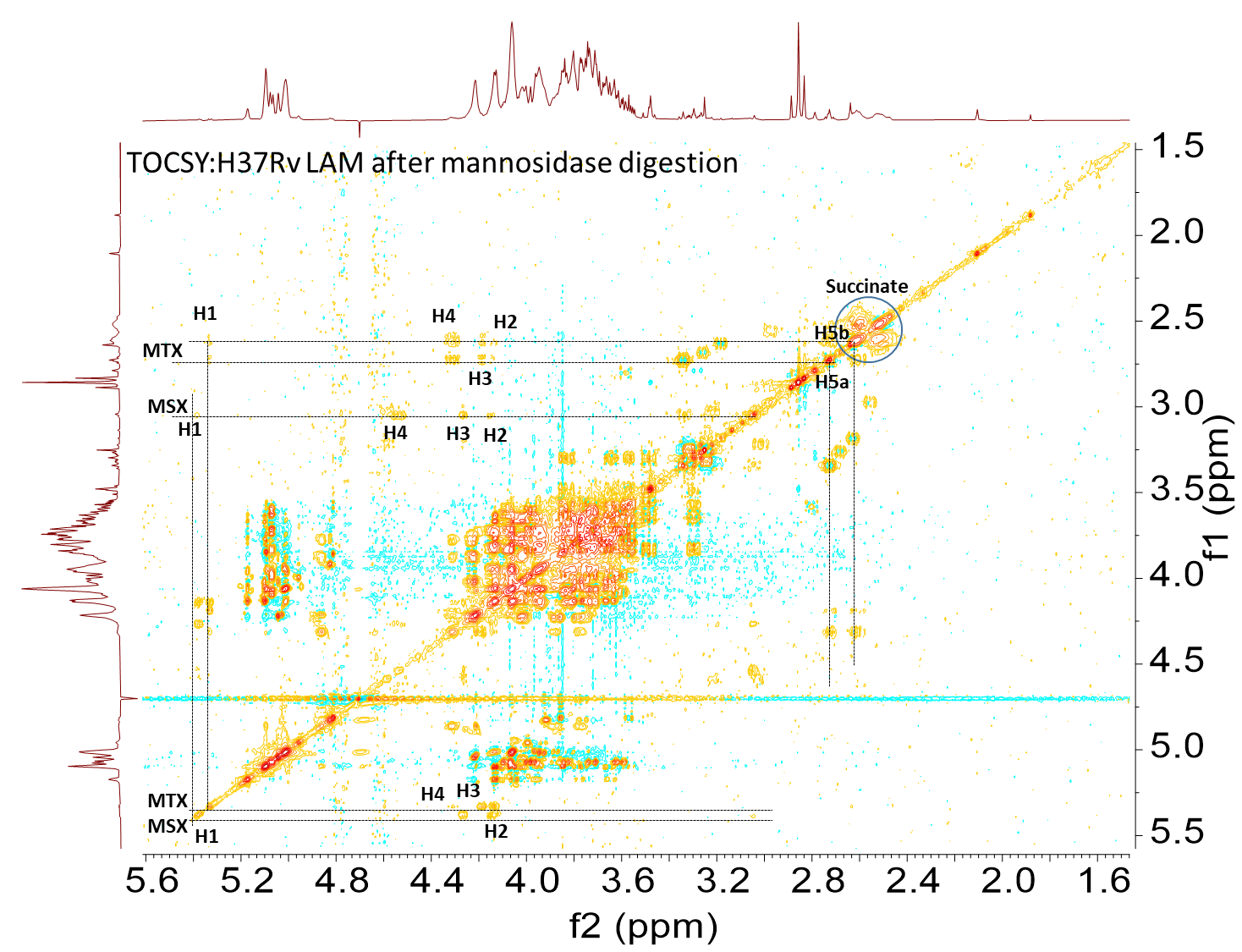


**Fig S7A:** ^1^H-^1^H Correlation (TOCSY, D_2_O, 256 scans, 500 MHz) NMR spectrum of H37Rv-LAM after α-Mannosidase digestion.


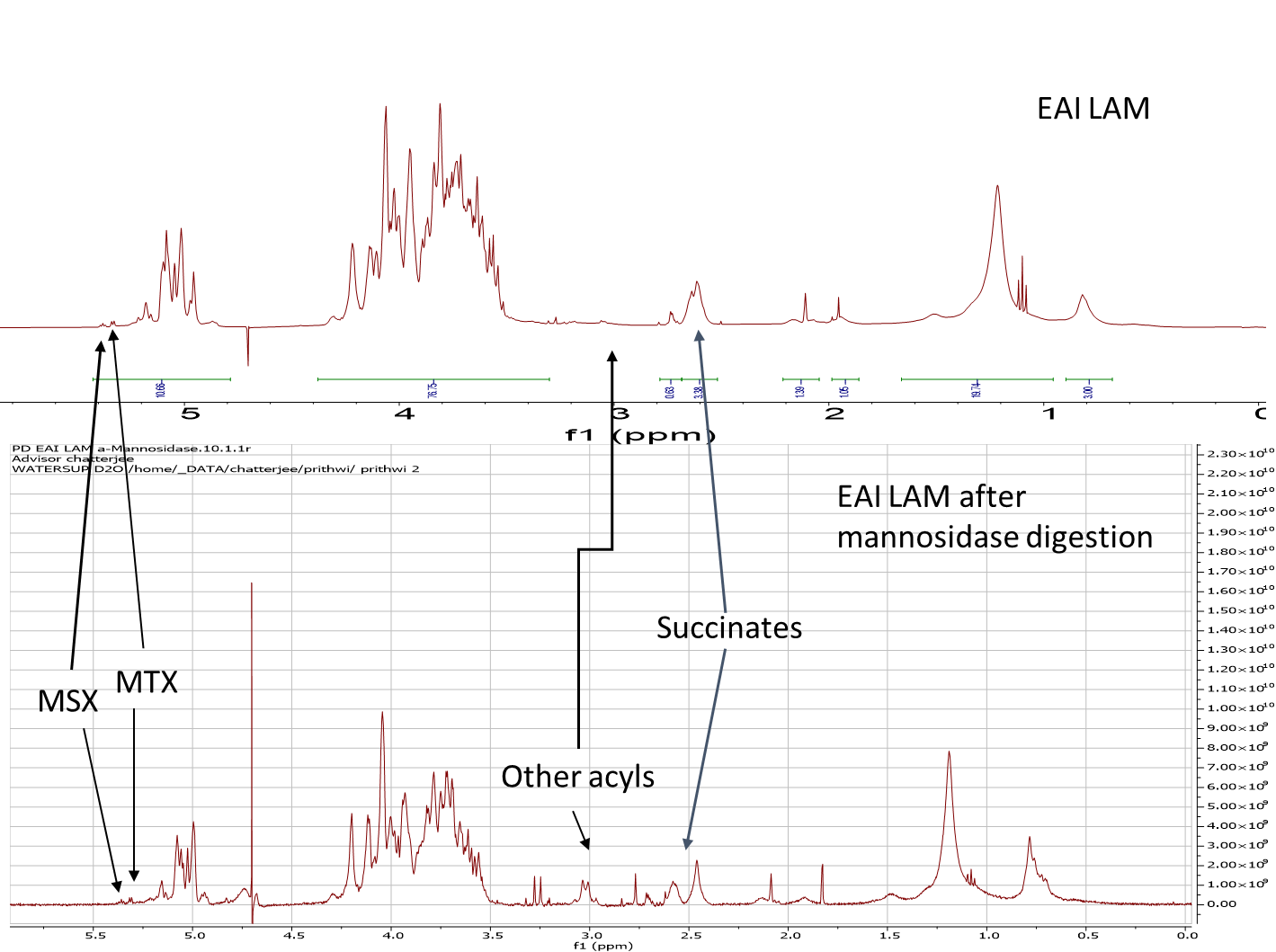
**Fig S7B:** ^1^H NMR (watersupp, D_2_O, 128 scans, 400 MHz) of EAI-LAM after α-Mannosidase digestion (Bottom); a comparison with intact-EAI-LAM (Top).


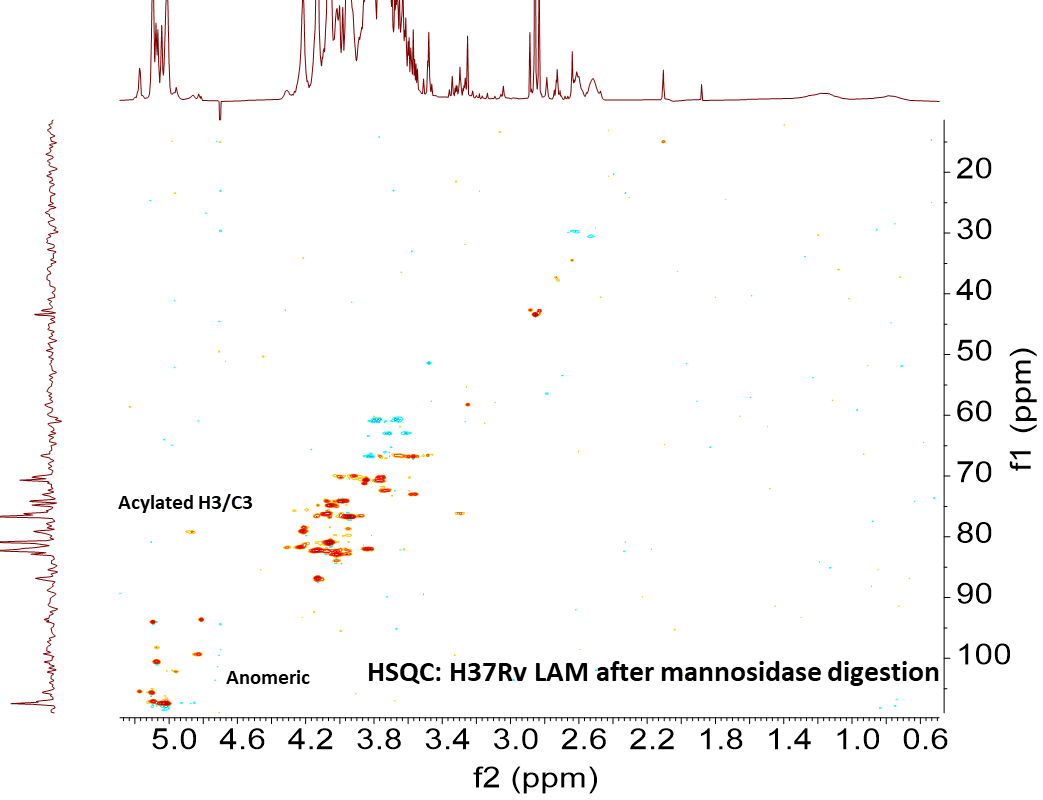


**Fig S7C:** ^1^H-^13^C Correlation (HSQC, D_2_O, 128 scans, 400 MHz) NMR spectrum of H37Rv-LAM after α-Mannosidase digestion.


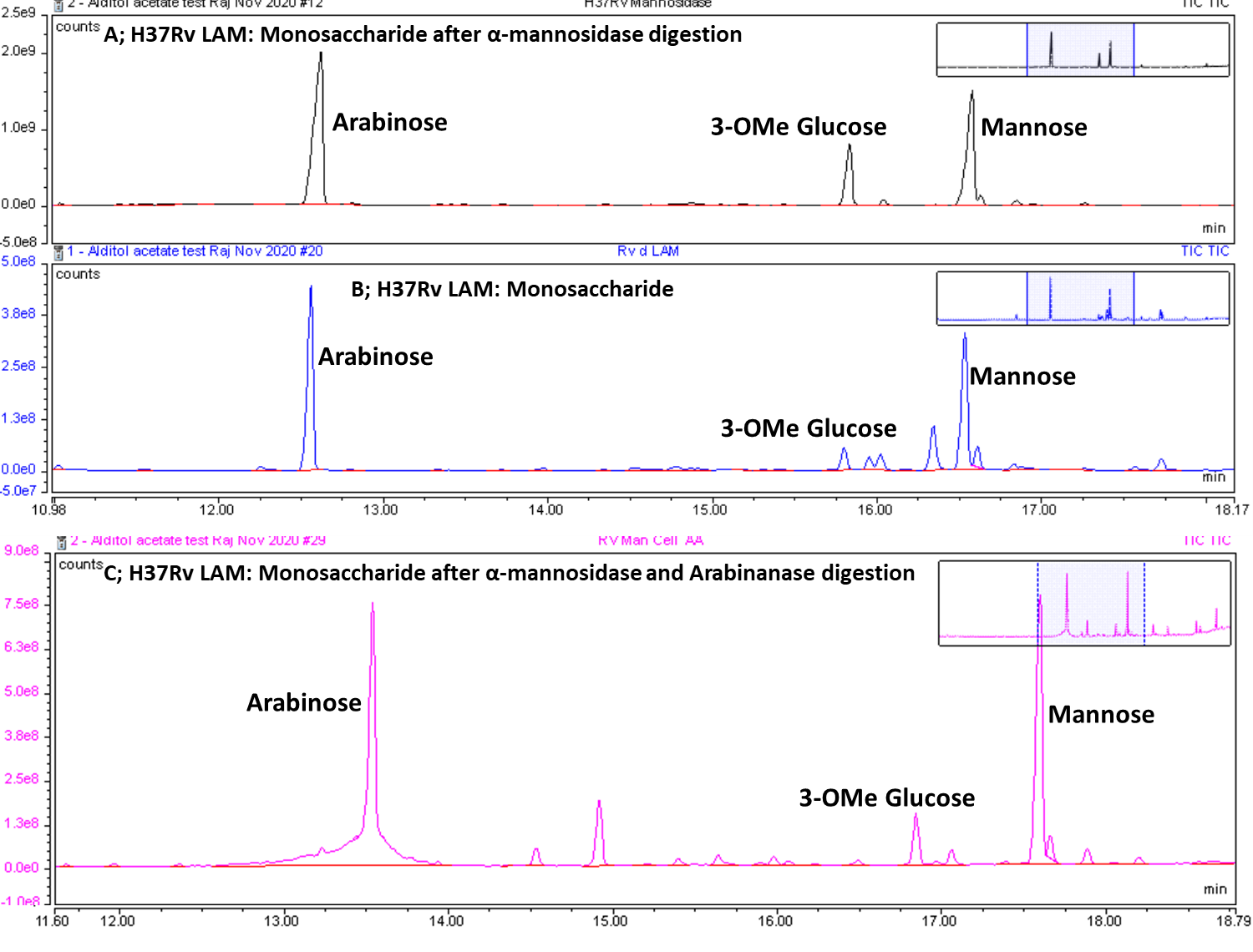


**Fig S8A:** Monosaccharide-Alditol acetate (GC/MS chromatogram, TIC) assay of Mannosidase digested H37Rv-LAM (Top), Intact-H37Rv-LAM (Middle) and Mannosidase-Arabinanase-digested H37Rv-LAM (Bottom).


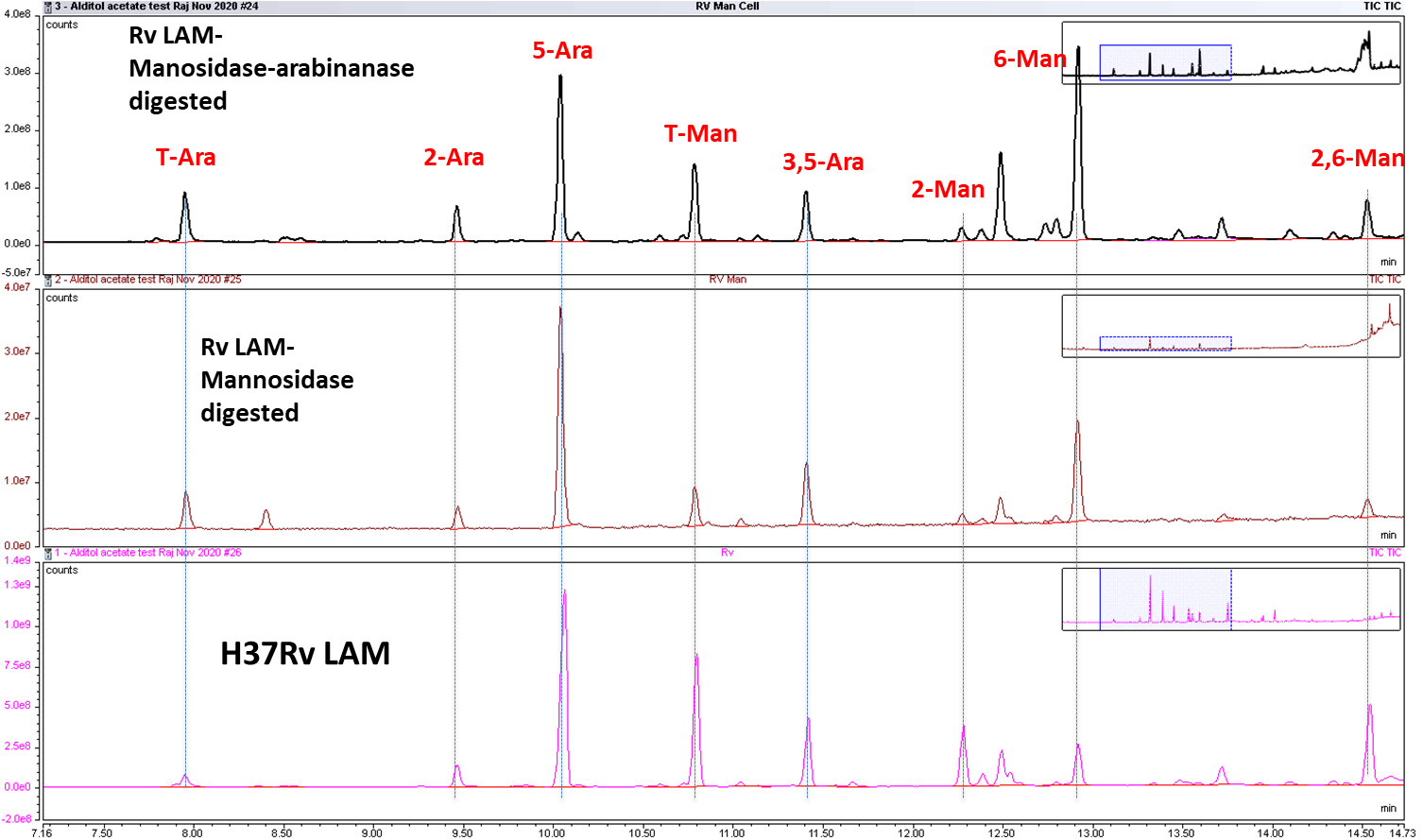


**Fig S8B:** Glycosidic-linkage (Permethylation-Alditol acetate) (GC/MS chromatogram, TIC) analysis of Mannosidase-Arabinanase-digested H37Rv-LAM (Top), Mannosidase digested H37Rv-LAM (Middle) and Intact-H37Rv-LAM (Bottom).

| **Linkage** | **HN878** | **EAI** | **IO** |
| --- | --- | --- | --- |
| **T-Ara** | 2.5 | 4.0 | 2.6 |
| **2-Ara** | 4.8 | 6.6 | 6.8 |
| **5-Ara** | 34.4 | 33.3 | 30.8 |
| **3,5-Ara** | 9.8 | 9.3 | 8.5 |
|  |  |  |  |
|  |  |  |  |
| **T-Man** | 18.7 | 18.8 | 20.6 |
| **2-Man** | 8.8 | 6.6 | 8.5 |
| **6-Man** | 5.8 | 6.3 | 7.0 |
| **2,6-Man** | 14.0 | 13.9 | 14.0 |

**Fig S8C: Relative percentage values of major glycosidic linkages of LAMs from clinical isolates.**

**
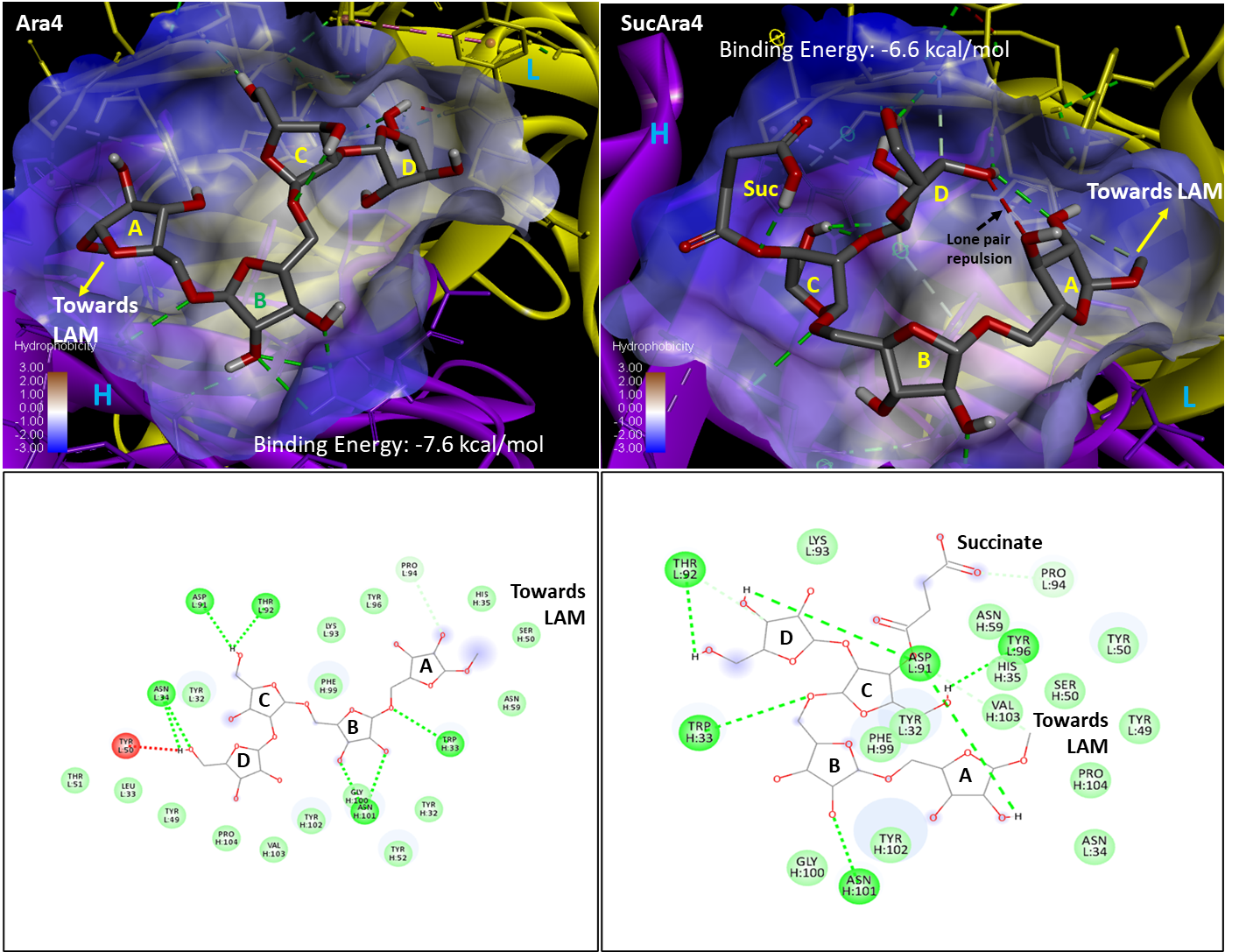
**

**Fig S9: Docking studies of Ara4 (Left Panel) and SucAra4 (Right Panel) with CS-35Fab (3HNT.pdb) on hydrophobic surface:** Purple ribbon: Heavy Chain and Yellow ribbon : Light Chain. Best pose (lowest score/binding energy and rmsd values) have been presented. Succinylation at the 3-position of ring-C-Ara*f* is unfavorable to binding compared to Ara6.


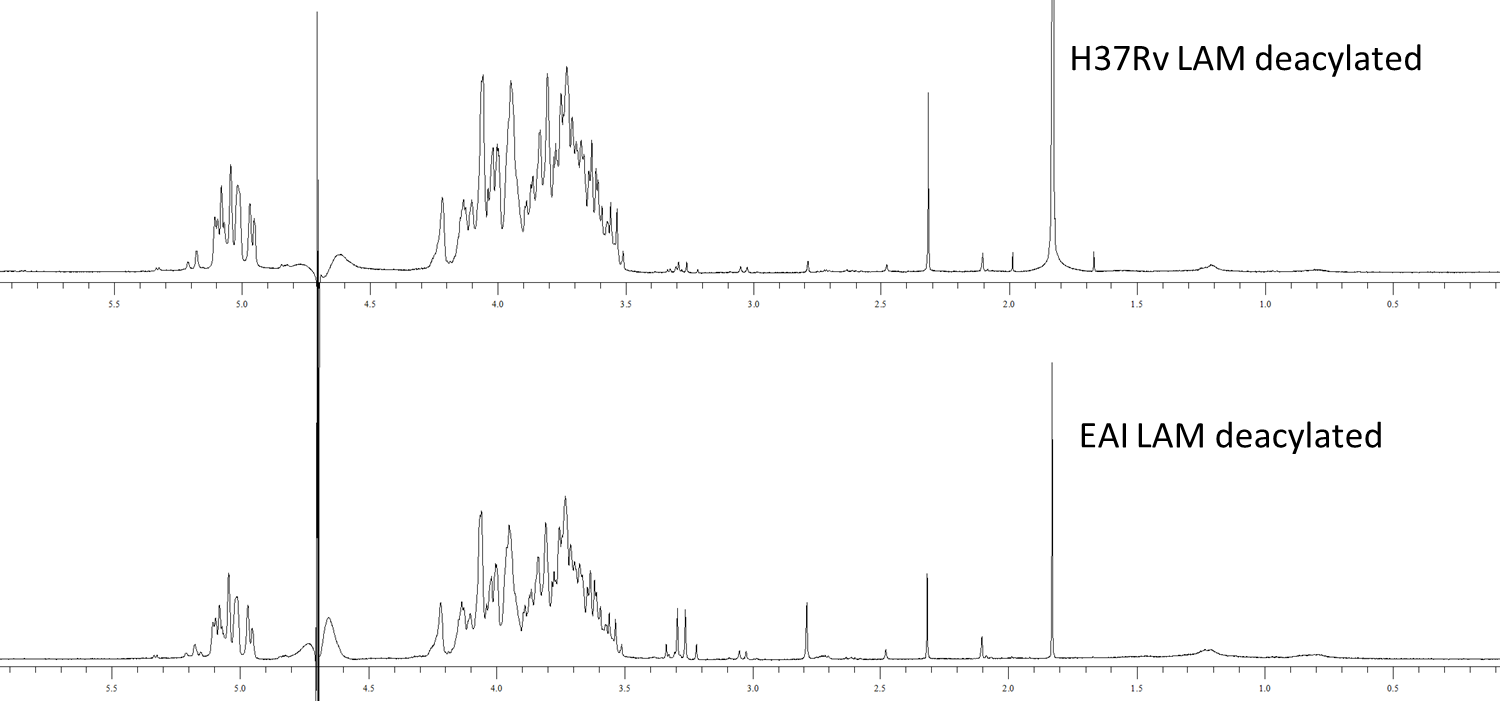
 **Fig S10:** ^1^H NMR (Watersupp, D_2_O, 256 Scans, 400 MHz) of deacylated (0.25 N Aq. NaOH; 37°C, 14 h) H37Rv-(Top) and EAI-LAM (Bottom).

**Materials and Methods**

*Preparation of Mycobacterium tuberculosis cell biomass*

Stocks of *M. tuberculosis* (*M.tb*) were originally received from the Trudeau mycobacterial culture collection (H37Rv), Centers for Disease Control and Prevention (CDC1551), the laboratory of Dr. James Musser (HN878) or the laboratory of Dr. Sebastian Gagneux (T17 – “IO” and 91-0079 – “EAI”). All growth conditions were performed by incubation at 37ºC. Seed stocks were made by colony isolation, subculture to 100 ml in 7H9-OADC, and freezing 0.8 ml samples in 20% glycerol. Genotyping was confirmed for the seed stocks by spoligotyping (performed through the TB Genotyping service at the Centers for Disease Control and Prevention). Working stocks (100 aliquots of 1 mL) were generated from one seed stock, and 3 working stocks were used to inoculate 10x100 cm 7H10-OADC plates with passage of bacterial lawns to 1L Fernbach flasks containing GAS media (38); these cultures were twice passaged to achieve 16 L cultures of each strain. Cells were harvested, washed with water, weighed, and cell biomass ranging from 70 – 80 g was inactivated by gamma irradiation (Ce137 irradiation, 2.4 MRad, adsorbed dose). Cells were lyophilized and stored at -80°C until use.

*Purification of Lipoarabinomannan*

Polar surface lipids were extracted from cells by thrice sequential incubation with chloroform:methanol:water (10:10:3) and cells broken as described previously (32). Insoluble cell material was removed by centrifugation at 27,000xg, 4℃ for one hour, and supernatant partitioned to extract the detergent, LAM enriched fraction.(32). Cold 200 proof ethanol was added to the detergent layers at 1:10 concentration, and samples incubated overnight at -20℃ to precipitate the LAM enriched material. The precipitate was collected by centrifugation at 27,000xg, 4°C for one hour, dried under nitrogen, weighed, and resuspended at 50mg/mL in endotoxin free water, and digested with proteinase K (final concentration of 0.1mg/mL) for 2 h at 60°C to remove any residual contaminating proteins.  The sample was dialyzed in running DI water using a 3,500 MWCO cassette, and SDS-PAGE gel and periodic silver stain were performed to confirm that the resulting LLP (LAM, LM, PIM_6_) sample does not contain residual proteins. For further purification, LLP was exchanged into LPS running buffer (0.2 M NaCl, 0.01 M Deoxycholic acid, 0.001 M EDTA in 0.01 M Tris, pH 8.0) and LAM, LM, and PIM_6_ separated using tandem size exclusion chromatography in LPS running buffer (HiPrep 26/60 Sephacryl-200 HR Column, 320 mL and HiPrep 26/60 Sephacryl-100 HR Column, 320 mL each) facilitated at 2.5 mL/min using a Waters 3525 High Pressure Liquid Chromatograph (HPLC). Fractions (2.5 mL) were collected and analyzed by SDS-PAGE. Pure LAM fractions were pooled and sequentially dialyzed using 12,000MWCO tubing against LPS dialysis buffer (minus deoxycholate), 1M NaCl, running DI water and lastly endotoxin free water. LAM was concentrated by lyophilization, then resuspended in minimal endotoxin free water to fully solubilize. LAM initially was qualified by ^1^H- NMR. For quantification of LAM, two methods were used. ImageJ (39, 40) was used to determine LAM concentration in mg/mL with a standard curve created by analysis of previously qualified and quantified LAM standard against the test sample resolved on a SDS-PAGE gel stained with periodate/silver-. For analysis using gas chromatography a GC-2014AFSC gas chromatograph (Shimadzu) was used to determine the ratios of the alditol acetate derivatives of mannose and arabinose and subsequent LAM concentration in mg/mL.

*Monosaccharide composition*.

LAM samples were hydrolyzed with 2 M trifluoroacetic acid. The resulting sugars were converted to alditol acetates and analyzed on a GC-MS using TRACE 1310 Gas Chromatograph (Thermo Scientific) equipped with a TSQ 8000 Evo triple-quad GC-MS/MS (Thermo Scientific). GC was connected to A DB5 Agilent capillary column (30 m × 0.25 mm i.d × 0.25-µm thickness, Phenomenex, CA, USA). The samples were injected (10 ng/µL), in the split-less mode at an initial temperature of 100 °C, under the constant flow of Helium carrier gas at a rate of 5 mL/min. Oven temperature gradient profile: begins at 100 °C, +20 °C/min to 150 °C, +5 °C/min to 240 °C (hold 3 min), and +30 °C/min to 300 °C (hold 5 min), with total acquisition time of 30 min. The mass spectrum was scanned from *m/z* 50-500; data analysis was performed using Chromeleon Chromatography data system software (Thermo Scientific). The data were quantitated by using response factors generated from authentic monosaccharides and *3-O-methyl* -glucose as an internal standard (41).

*NMR experiments*

All NMR spectra were recorded with ~5 mg LAM in D_2_O (0.56 mL) at 25°C. All PRESAT ^1^H NMR was recorded on a Bruker Neo 400 MHz NMR instrument. ^1^H-^13^C correlation (C2HSQC) NMR spectra were recorded with 128 scans and relaxation delay 1.2 s on Bruker Neo 400 MHz NMR instrument. All chemical shifts are based on the reference to the HOD peak at 4.74 ppm. The default Bruker parameters were used for recording spectra. TOCSY (^1^H-^1^H correlation) NMR spectra were recorded with 256 scans on Varian Innova 500 MHz instrument. All chemical shifts are based on the reference to the HOD peak at 4.64 ppm. The default Varian parameters were used for recording spectra. Spectra were processed using MestReNova x64.

α *-Mannosidase treatment conditions*

A solution of LAM (1 mg/0.2 mL) was treated with 0.085 mL (5 units) of α-Mannosidase (Jack Beans; Sigma; 3.5 mg/mL; pH 5.5) and incubated at 37°C (water bath) for 20h. The reaction mixture was then frozen at -80°C for 2h. It was subjected to 3X freeze-thaw cycles and then heated at 80°C for 15 min. The reaction mixture was subsequently purified over Biogel-P4 column (30 cm X 1 cm).

*Endoarabinanase digestion to release oligoarabinosides*

LAM (100-200 µg) from each strain was digested with *endo*arabinanase as previously described (21) and the released arabinan fragments were purified from the resistant core by nanosep 3K Omega (Pall Corporation) and centrifuging at 14,000xg for 20 mins at room temp. An aliquot of samples was analyzed by SDS-PAGE followed by Periodic acid silver staining to ensure complete digestion of LAM.

The flow through containing the released oligoarabinosides were analysed by LC/MS in their native form. This material was further analyzed on Bruker maxi plus II Q-TOF instrument.

*Immunoassays by Indirect and Capture ELISA*

Indirect ELISA

Indirect ELISA was carried out as previously described (42) with some modifications. LAM were prepared in the coating buffer (0.05M sodium carbonate and sodium bicarbonate, pH 9.6) at the final concentration of 1µg / mL and applied to the 96 well plate (Corning,Costar) at 4^o^C overnight. Non- specific antibody binding sites were blocked by adding the blocking buffer (1% BSA (Sigma Aldrich) in 1X PBS to the antigen coated wells. Purified CS35 was used at a starting concentration of 5µg/mL and serially diluted two-fold to derive a concentration curve and added to the wells and incubated at room temperature for 90 min. The plates were then washed with the wash buffer (1X PBS containing 0.05% Tween-80) and incubated at room temperature for another 90 min with the anti-mouse IgG alkaline phosphatase conjugated secondary antibody (1:2500 dilution) in wash buffer. The plates were washed and the alkaline phosphatase activity measured by addition of p-nitrophenyl phosphate (pNPP) (Kirkegard and Perry Laboratories) as a substrate. The optical density was measured at 405nm.

Capture ELISA

Assay was performed as previously described elsewhere with modifications (10). A 96 well polystyrene high binding microplate (Corning, Costar) was coated with CS35 at a concentration of 10 µg/mL in 1xPBS) and incubated at 4^o^C overnight. After overnight incubation, the antibody coated plates were brought to room temperature and the plates were blocked using 1% BSA in 1X PBS for 60 min at room temperature. LAM and derivatives were prepared in 1X PBS at the final concentration of 12.5ng/mL and serially diluted two-fold and applied in duplicate to the plate/s. The plates were incubated at room temperature for 90 min followed by washing (1X PBS containing 0.05% Tween-80). The plates were then incubated with the detection antibody (A194 hu mAb) conjugated with biotin at the final concentration of 250ng/mL, at room temperature for 90 min. Following a third wash, 1:200 dilution of Streptavidin-Horseradish Peroxidase (R&D Systems) was added to the plates and incubated for 25 min at room temperature. After the final wash, Ultra TMB ELISA chromogenic substrate (Thermo Scientific) was added to the plates, incubated at room temperature till the color development. The reaction was stopped by adding 2M Sulphuric acid and the optical density measured at 450nm.

*Conditions for LC/MS*

Structural elucidation was carried out via ultraperformance liquid chromatography (UPLC) separation on a Waters Acquity UPLC H-Class system inline with a Bruker MaXis Plus quadrupole time-of-flight (QTOF) mass spectrometer (MS). Separation was performed in gradient mode with a Waters Atlantis T3 3.0 μm column (2.1 × 150 mm) at 40 °C. Mobile phase components were 10 mM ammonium acetate in water (A) and 10 mM ammonium acetate in acetonitrile (B). The flow rate was 0.3 mL/min. The proportion of acetonitrile was held at 0% for 1 min, then increased from 0% to 95% for 9 min, and held at 100% for 3 min. The post-time was 7 min, and the injection volume was 4 μL. For full scan experiments, data were acquired in the negative electrospray ion (ESI) mode with a mass-to-charge ratio (m/z) range of 110−4000 at 1 Hz scan rate. Source settings were as follows: capillary voltage, 3500 V; end-plate offset, 500 V; nebulizer gas pressure, 3 bar; drying gas flow, 10 L/min; drying temperature, 300 °C. For MS/MS experiments, the sources settings noted above were the same except for the 2−6 min time segment; the MS was in multiple reaction monitoring (MRM) scan mode with collision-induced dissociation (CID) energies of 40 eV and 60 eV on target masses with *m/z* width of 10 Da. Internal instrument mass-scale calibration was performed in enhanced quadratic mode during chromatographic dead time by infusing the Agilent ESI-L low concentration tuning mix. Instrument controls were performed via the Bruker HyStar v4.1 software package. Data were processed using Bruker Compass 2.0 Data Analysis 4.4 software.
